# In vitro assessment of the genotoxicity and immunotoxicity of treated and untreated municipal effluents and receiving waters in freshwater organisms

**DOI:** 10.1007/s11356-023-26845-1

**Published:** 2023-04-15

**Authors:** Ève A. M. Gilroy, Christine Kleinert, Émilie Lacaze, Sheena D. Campbell, Sara Verbaan, Chantale André, Kara Chan, Patricia L. Gillis, Joel S. Klinck, François Gagné, Michel Fournier, Shane R. de Solla

**Affiliations:** 1 Aquatic Contaminants Research Division, Environment and Climate Change Canada, Burlington, ON Canada; 2 Green House Science, Burlington, ON Canada; 3 Ecotoxicology and Wildlife Health Division, Environment and Climate Change Canada, Burlington, ON Canada; 4 Institut National de La Recherche Scientifique-Centre Armand-Frappier Santé Biotechnologie, Laval, QC Canada; 5 Aquatic Contaminants Research Division, Environment and Climate Change Canada, Montréal, QC Canada; 6 Redeemer University, Ancaster, ON Canada

**Keywords:** Genotoxicity, Immunotoxicity, Wastewater treatment, Freshwater mussels, Fish

## Abstract

Municipal wastewater effluent is one of the largest sources of pollution entering surface waters in the Laurentian Great Lakes. Exposure to wastewater effluent has been associated with impaired immune systems and induction of genotoxicity to aquatic animals. Due to habitat degradation and environmental pollution linked to industrial development and population growth, several regions of the Great Lakes have been designated Areas of Concern (AOCs). In this study, we assessed the effect of extracts of sewage influent, (treated) effluent and receiving surface waters from the Hamilton Harbour AOC and the Toronto and Region AOC (Ontario, Canada) on the phagocytic immune response of rainbow trout (*Oncorhynchus mykiss*) kidney leukocytes and the genotoxicity (DNA strand breaks) of these extracts on freshwater mussel (*Eurynia dilatata*) hemocytes. We identified and quantified numerous chemicals present in the various samples extracted for exposure. In freshwater mussels, extracts from Hamilton Harbour AOC induced DNA damage with the most frequency (12 out of 28 samples) regardless of sample type, reflecting past and present industrial activities. In contrast, extracts from Toronto and Region AOC induced DNA damage infrequently (2 out of 32 (summer) and 5 out of 32 (fall) samples, respectively) and from different WWTPs at different times. None of the extracts induced any significant effect on phagocytosis of rainbow trout kidney leukocytes. The present study indicates that despite overall improvements to effluent quality, treatment of influent by WWTPs may not result in a corresponding improvement of the genotoxicity of effluents. In vitro bioassays are useful and cost-effective rapid-screening tools for preliminary assessments of contamination of aquatic ecosystems.

**Supplementary Information:**

The online version contains supplementary material available at 10.1007/s11356-023-26845-1.

## Introduction

Following the expansion of industrial development and rapid population growth of the last century, many regions of the Great Lakes have been greatly compromised by habitat degradation or loss due to anthropogenic factors such as contamination and eutrophication (Environment Canada 2003). Under the 1987 protocol of the International Joint Commission, 43 of these regions have been designated Areas of Concern (AOCs) due to the impairment of aquatic ecosystems and resulting restrictions on economic or recreational activities. There are two AOCs located in and near the greater Toronto area (population ca. 6,202,000 in 2021) in southern Ontario. The Toronto and Region AOC receives drainage from 6 watersheds draining regions of urban, agricultural, and industrial land use. Contamination by combined sewage overflow, road runoff, and industrial and urban effluent has contributed to habitat degradation, restrictions on fish and wildlife consumption, beach closures, restrictions on dredging activities, and degradation of fish and wildlife populations and habitat (Environment Canada 2011a). The Hamilton Harbour AOC, located between the cities of Hamilton (population ca. 570,000 in 2021) and Burlington (population ca. 187,000 in 2021), has also been impaired by intensive industrial and urban development around its shores. Hamilton Harbour supports a large concentration of heavy industry and receives the discharge from three wastewater treatment plants and urban runoff from the cities of Hamilton and Burlington. Sediments in some areas of the harbor are heavily contaminated with metals, polychlorinated biphenyls (PCBs), and polycyclic aromatic hydrocarbons (PAHs) released by past and ongoing industrial practices (Environment Canada 2011b; Milani and Grapentine 2016; Graham et al. 2017).

The primary goal of wastewater treatment plants (WWTPs) is to treat domestic sewage to ensure that the receiving surface waters are of sufficient quality to maintain both healthy aquatic ecosystems and unhindered recreational and economic uses. The prime means of maximizing environmental quality of the effluent is through minimizing the discharges of suspended solids, phosphorus, nitrogen, and biological oxygen demand (BOD), mostly based on meeting Provincial or Federal Water Quality Guidelines (Ontario Ministry of the Environment and Energy 1994). Historically, WWTPs were not designed to eliminate persistent organic compounds, and thus compounds such as pharmaceuticals or halogenated organic compounds are common in wastewater effluent (Ohe et al. 2004; Hébert et al. 2008; Holeton et al. 2011). Combined sewer systems, which collect runoff from the urban landscape, are diverted to WWTPs, carrying significant loadings of sediments, road salts, metals, oils and grease, and PAHs (Phillips and Chalmers 2009; Kiesling et al. 2019). Lastly, WWTPs can release by-products or degradation products created from water treatment processes, such as chloramines and metabolites of pharmaceuticals (Environment Canada 2002). Consequently, municipal wastewater effluents are significant point sources for a broad suite of these substances to surface waters in the Great Lakes Basin.

Effluent from WWTPs can induce a variety of effects in aquatic animals, from genomic and physiological responses to alterations at the population and ecosystem levels. Effluent exposure can disrupt endocrine function and impair reproduction (Jobling et al. 1998; Tetreault et al. 2012; Fuzzen et al. 2016), alter immune response (Farcy et al. 2011; Gillis 2012; Jasinska et al. 2015), and induce oxidative stress (Gillis et al. 2014) in aquatic vertebrate and invertebrates. Mussel communities have suffered both local extirpations and dramatic reductions in population size downstream of WWTP outfalls (Horne and McIntosh 1979; Goudreau et al. 1993; Nobles and Zhang 2015; Gillis et al. 2017a). Exposure to contaminants found in wastewater effluents can induce immune responses in mussels (Gillis et al. 2014) and can increase the susceptibility of aquatic invertebrates to pathogens, by suppressing or impairing phagocytic, cytotoxic, or inflammatory responses as part of the innate immune responses (Akaishi et al. 2007; Coray et al. 2007; Gillis et al. 2014). The complex interactions of immune, neuroendocrine, and reproductive factors often make it difficult to establish direct causal links between functional alterations and changes in resistance to disease.

Phagocytosis is an important mechanism of innate immunity present in both aquatic vertebrates and invertebrates (Rinkevich 1999; Buchmann 2014), where foreign particles and pathogens are engulfed and destroyed intracellularly. Contaminant-induced changes in phagocytic efficiency have been reported in organisms exposed to a number of contaminants, including metals (Fournier et al. 2000), PAHs (Reynaud and Deschaux 2006), pharmaceuticals (Lacaze et al. 2015), and municipal effluents (Hébert et al. 2008; Farcy et al. 2011; Gagné et al. 2013; Gust et al. 2013; Lacaze et al. 2017).

Municipal effluents are also known to induce genotoxicity in aquatic animals. DNA mutations, if not repaired, can initiate a cascade of biological effects from the cellular to the population level. DNA-damaging agents can have a significant ecological relevance since they are implicated in many pathological processes and can exert transgenerational effects (Sánchez-Argüello et al. 2012). For example, genotoxicity can lead to a reduction in population size or structure by affecting fertility rates, gamete loss, impaired development, embryonic mortality, and heritable mutations in freshwater invertebrates and fish (Lewis and Galloway 2009; Lacaze et al. 2011; Santos et al. 2012).

The goals of the present study were to use in vitro assays to provide insight into the sub-lethal effects of urban influents, effluents, and receiving surface waters on aquatic species, to use these assays to assess the effectiveness of WWTPs at reducing exposure to harmful contaminants, and to evaluate their usefulness as rapid-screening tools for assessing the toxicity of wastewater effluent and surface waters. Using two in vitro assays, we assessed the genotoxic and immunotoxic potential of treated and untreated wastewater collected in two urban AOCs. To examine immunotoxicity of wastewater, we quantified the phagocytic activity of kidney leukocytes from rainbow trout (*O. mykiss*). To determine if exposure to wastewater effluent induces DNA strand breaks, we used the single cell gel electrophoresis assay (i.e., comet assay), with freshwater mussel hemocytes (*Eurynia dilatata*). Additional samples were also collected in parallel for chemical analysis of a suite of chemicals of concern, to relate to the results of the in vitro assays. We predicted that influent would induce DNA damage and impair the immune response of mussels and fish, to a greater degree than either effluent or surface waters from environments that receive wastewater effluent, and that dilution of wastewater effluents would reduce the toxicity.

## Materials and methods

### Water sample collection and preparation

**Fig. 1:**
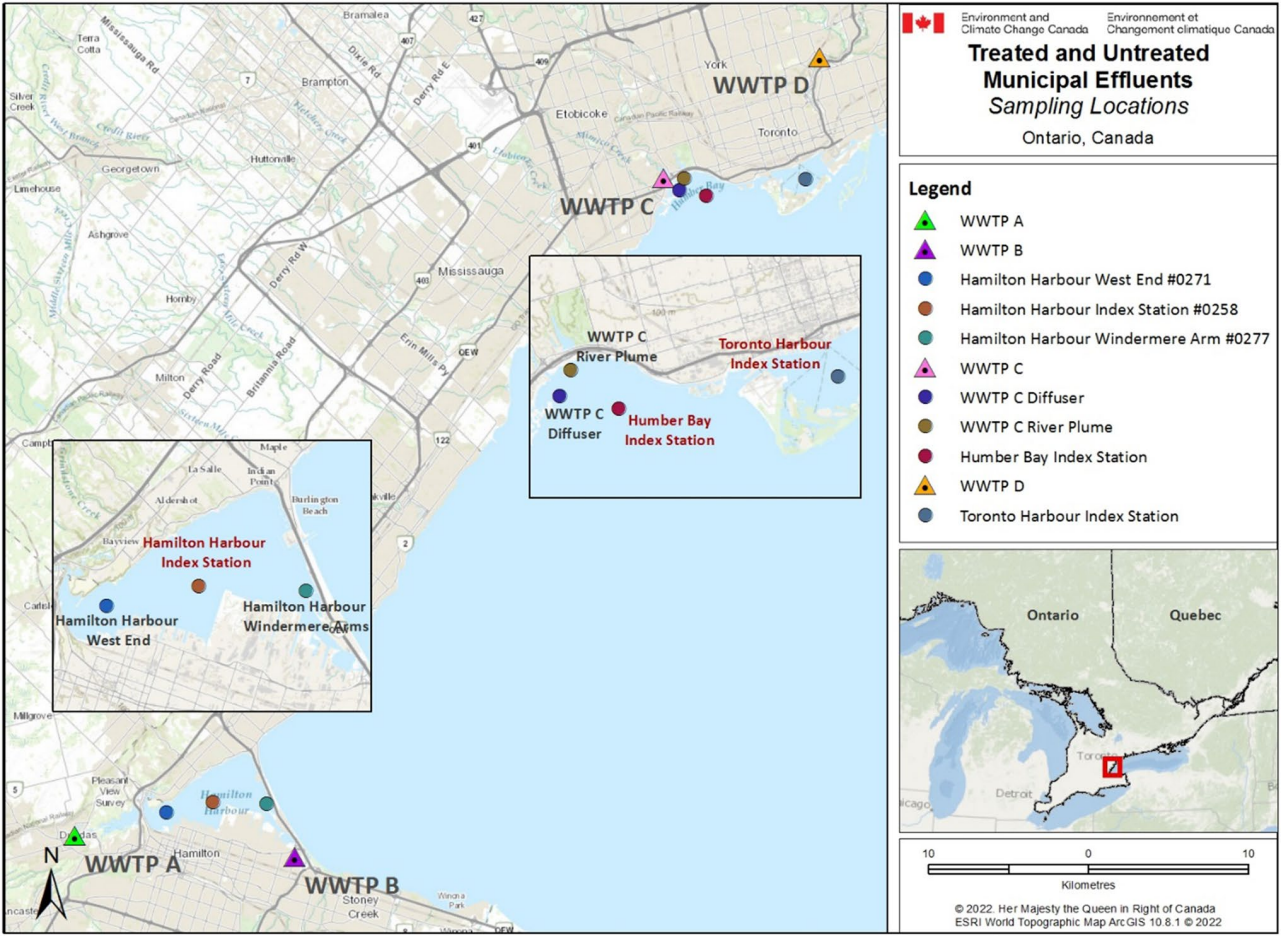
Map of the sampling locations for the assessment of the immunotoxicity and genotoxicity of treated and untreated municipal effluents and surface waters in two Canadian Areas of Concern in Lake Ontario, ON, Canada

Surface water samples were collected from three locations in Lake Ontario: Hamilton Harbour in the Hamilton Harbour AOC, and Toronto Harbour and Humber Bay, in the Toronto and Region AOC (Fig. 1). Wastewater samples (influent and effluent) were collected from four WWTPs within these locations: WWTP A and WWTP B in Hamilton Harbour AOC, and WWTP C and WWTP D in the Toronto and Region AOC. All samples were collected between November 2014 and October 2015 (Table 1). Surface water grab samples were collected from seven sites within these three locations: Hamilton Harbour (West End, Windermere Arm, Hamilton Harbour Index Station), Humber Bay (near the Diffuser, River Plume, Humber Bay Index Station), and Toronto Harbour (Toronto Harbour Index Station). To assess the seasonal influence of the watershed, the samples from the Toronto AOC (Humber Bay and Toronto Harbour) were collected in the spring and the fall, during both high and low influent flow rates, respectively. Wastewater and surface water samples for were collected by the Ontario Ministry of the Environment and Climate Change (OMECC) and shipped on ice to Center Saint-Laurent (Environment and Climate Change Canada, Montréal, QC) by overnight courier, where they were frozen at -20 °C pending extraction. Samples were thawed and filtered through a glass microfiber filter (GF/C, 1.2 µm pore size, Whatman, Mississauga, ON, Canada) to remove suspended solids. The filtrates were then fractionated on a reverse phase 6 mL/500 mg Chromabond C18 SPE cartridge (Macherey–Nagel, Düren, Germany) activated with 6 mL of Milli-Q water. After sample loading at a flow rate of 2–4 mL/min, cartridges were washed with 6 mL Milli-Q water, and analytes were eluted with analytical-grade ethanol (EtOH) (Absolute; Sigma-Aldrich, Oakville, ON, Canada) to produce a final concentrate of 1000 × . Sample extracts were divided, and a portion of each was shipped on ice to the Canada Center for Inland Waters (Environment and Climate Change Canada (ECCC), Burlington, ON). Samples were frozen at -20 °C pending analysis. Prior to experimentation, the extracts were brought to room temperature and diluted in freshwater phosphate-buffered saline (PBS—fish) or 25% PBS (mussels). The genotoxicity method was validated using controls without EtOH, solvent controls at 0.1% EtOH, and positive controls (0.25 mM hydrogen peroxide (H_2_O_2_), known to induce DNA damage); all subsequent exposure solutions contained 0.1% EtOH and a positive control.

**Table 1:** List of samples collected by the Ontario Ministry of the Environment, Conservation and Parks for the assessment of the immunotoxicity and genotoxicity of treated and untreated municipal effluents and surface waters in two Canadian Areas of Concern in Lake Ontario, ON, Canada. Wastewater treatment at all wastewater treatment plants (WWTPs) includes conventional activated sludge (secondary treatment). Treatment at WWTP A also includes sand filtration (tertiary treatment)

Sample location	Sample identification	Collection dates
Hamilton Harbour	WWTP A—influent	Nov 2014
	WWTP A—effluent	
	WWTP B—influent	
	WWTP B—effluent	
	Hamilton Harbour West End	
	Hamilton Harbour Index Station	
	Hamilton Harbour Windermere Arm	
Humber Bay	WWTP C—influent	Jun 2015 Oct 2015^a^
	WWTP C—effluent	
	WWTP C—diffuser	
	WWTP C—Humber River Plume	
	Humber Bay Index Station	
Toronto Harbour	WWTP D—influent	Jun 2015 Oct 2015^a^
	WWTP D—effluent	
	Toronto Harbour Index Station	

^a^Samples were analyzed using the mussel comet assay only

### Freshwater mussel assays

#### Freshwater mussel handling

Adult freshwater mussels (*Eurynia dilatata*, common name Spike; approximately 60–90 mm) were collected from a site in the Grand River watershed known to have a large stable population (Gillis et al. 2017b) and held in ECCC’s Aquatic Life Research Facility in Burlington, ON, Canada at 12 ± 2 °C in a flow-through system with dechlorinated Burlington city tap water and fed a commercial shellfish diet (Shellfish Diet 1800®, Reed Mariculture, Richmond Hill, ON) twice per week. Mussels used for exposures conducted in September and October were collected in July (holding time of 10–13 weeks). Mussels used for exposures conducted in March were collected in October (holding time of 21–23 weeks). On the day of the experiment, mussels were transferred to an aerated cooler.

#### In vitro exposures

Hemolymph was collected from the adductor muscle of adult mussels using 5 mL syringes equipped with a 22G needle and kept on ice. The hemolymph from two or three mussels was pooled when the volume was not sufficient for use with every extract. Viability and cell density were assessed using the Trypan Blue exclusion method to select hemolymph with cell viability > 90% and cell density > 2.5·10^5^ cells/mL for use in the experiments. Four hemolymph replicates (hemolymph from four different mussels or groups of mussels) were used to assess the genotoxicity of the water extracts from Hamilton Harbour, due to limited extract availability; eight replicates were used to assess the genotoxicity of the samples from the Toronto and Region AOC.

The in vitro exposures followed the methods described by Lacaze et al. (2015), with some modifications. Briefly, 150 µL of sample extract prepared in 25% PBS was added to 150 µL of hemolymph, for final exposure concentrations of 100%, 50%, 25%, and 12.5% of reconstituted sample, 0.1% EtOH (solvent control, SC) and 0.25 mM H_2_O_2_ (positive control). Cells were exposed to the sample extracts in 96-well microplates for 4 h at 20 °C in the dark, under gentle agitation (150 rpm). Four to eight replicates per concentration and per sample were used. Method validation for the use of EtOH as a solvent control and H_2_O_2_ as a positive control is included in the Supplementary Information (Figure S1). The experimental design is summarized in Table S1.

At the end of the exposure, the viability and cell density of hemolymph from each treatment was assessed by flow cytometry (Guava, Luminex Corp., Austin, TX, USA). The percentage of dead cells was determined by dilution with a Guava Viacount reagent (Sigma-Aldrich, Oakville, ON, Canada). For each sample, 1,000 events were acquired. The cell population was electronically gated in a forward and side scatter density dot plot and the fluorescence frequency distribution histogram was obtained. Data collection and analysis were performed with the Guava Viacount software (ver. 2.5.2, EMD Millipore, Billerica, MA, USA). The results were expressed as a percentage of viable cells.

#### Genotoxicity assay (comet assay)

Ten microliters of each cell suspension was combined with 90 µL of 1% low-melting agarose, spread onto a 20-well comet slide (Trevigen, Gaithersburg, MD, USA), and processed under alkaline conditions according to the manufacturer’s instructions. Briefly, the slides were immersed in lysis solution at 4 °C for 1 h, in alkaline unwinding solution (Trevigen, Gaithersburg, MD, USA) at room temperature for 1 h, and then submitted to alkaline electrophoresis (21 V, 220 mA) for 40 min. After rinsing in water and EtOH, the slides were dried at 60 °C, stained with SYBR® Gold (Thermo Fisher Scientific, Burlington, ON, Canada), and visualized by fluorescence microscopy. Pictures of 50 cells per sample (or maximum number) were processed using a comet application in Northern Eclipse (Empix Imaging, Cheektowaga, NY, USA). When the results from specific replicates did not provide a sufficient amount of cells for scoring, the entire replicate was removed from the analysis. The percentage of DNA in the comet tail (% DNA in tail) was selected as the most reliable and meaningful measurement.

### Rainbow trout assays

#### Fish handling

Female rainbow trout (*Oncorhynchus mykiss*, 78 ± 12 g (mean ± standard deviation, *n* = 16)) were acquired from a local hatchery (Pisciculture des Arpents verts, St-Edwige, QC), transported to Center Saint-Laurent (Montréal, QC), and were acclimatized for 2 weeks in 300 L tanks filled with aerated, UV-treated, and charcoal-filtered City of Montreal tap water at 15 °C. Fish were fed daily with commercial trout feed (Nutra Fry, Skretting, St. Andrews, NB), until the day before euthanasia. During each dissection, four trout were euthanized in 100 mg/L tricaine methanesulfonate (MS-222, Sigma-Aldrich, ON, Canada) buffered with 100 mg/L NaHCO_3_ for 5 min at 15 °C in accordance with the Canadian Council on Animal Care methods.

#### Leukocyte preparation

Fish leukocytes were prepared following Müller et al. (2009), with some modifications. The anterior kidney of each fish was dissected aseptically and homogenized with a 2 mL glass grinder (Wheaton Scientific, NJ, USA) containing 1 mL sterile RPMI-1640 cell culture medium (Bio Media, Toronto, ON, Canada) supplemented with 10 IU/mL heparin (Organon Teknika, Durham, NC, USA), 10 mM HEPES (4-(2-hydroxyethyl)-1-piperazineethanesulfonic acid), 100 IU/mL penicillin, 100 mg/mL streptomycin, and 10% (v/v) fetal bovine serum (Bio Media, Toronto, ON, Canada). The cellular suspension was transferred to a sterile 15 mL conical polypropylene tube, and the final volume was adjusted to 5 mL with RPMI-1640 cell culture medium. The cell suspension was laid over 5 mL of Ficoll gradient medium (Cedarlane Laboratories, Burlington, ON, Canada) and centrifuged at 400 × g for 30 min. Leukocytes between the two layers were removed by aspiration using a sterile Pasteur pipette and transferred to a sterile conical polypropylene tube. The cells were washed twice in PBS and resuspended in heparin-free complete RPMI. Cell density and viability was evaluated with a hemocytometer using the Trypan Blue exclusion method. Cell density was adjusted to 8 × 10^5^ cells/mL; cell viability before exposure was 98 ± 1%.

#### In vitro leukocyte exposure

Cells were exposed to water extracts for 24 h at 15 °C in the dark, under gentle agitation (120 rpm), by replacement of half of the initial culture medium by the organic extracts. Cells at a final cell density of 4 × 10^5^ live cells/mL were exposed in 24-well plates to increasing concentrations of influent, effluent, and surface water sample extracts (12.5%, 25%, 50%, and 100%). Controls without EtOH and solvent controls at 0.1% EtOH were used. Three replicates per concentration and per sample were used. The experimental design is summarized in Table S1.

At the end of the exposure, 4 μL propidium iodide (PI, 100 μg/mL) was added to the cell suspension, and leukocyte mortality was evaluated by flow cytometry (FACSCalibur, Becton Dickinson, San Jose, CA, USA). For each sample, 5,000 events were acquired. Data collection and analysis were performed with the CellQuest Pro software (Version 4.0.1). The results were expressed as the percentage of viable cells.

#### Immunological assessment

For the phagocytosis measurement, we adapted a method developed by Evariste et al. (2018). After a 4 h exposure, 500 µL of cell suspension was incubated with carboxylate-coated fluorescent latex beads (Molecular Probes Inc., Eugene, OR, USA) in sterile 5 mL tubes. The phagocytic activity (cells having engulfed ≥ 1 bead) and efficiency (cells having engulfed ≥ 3 beads) were measured after 20 h by flow cytometry (FACSCalibur, Becton Dickinson, San Jose, CA, USA). For each sample, 10,000 events were acquired. All measured values of the % of cells having engulfed ≥ 1 or 3 beads were then transformed relative to their respective 0.1% EtOH solvent control (that was set to 100%).

### Chemical analysis

Chemical analysis of the influent, effluent, and surface water samples was conducted at the Ontario Ministry of Environment, Conservation and Parks (OMECP) Laboratory Services Branch in Toronto, Ontario. Accredited methods, practical quantitation limit, and method detection limits for these analyses are listed in Tables S2, S3, and S4 (Supplementary Information). The following categories of target parameters were analyzed: conventional parameters (pH, alkalinity, conductivity, solids (total, suspended, dissolved), chemical oxygen demand, nitrogen (ammonia + ammonium, nitrite, nitrate + nitrite), and phosphorus (phosphate)) using MECP Methods 3218, 3188, 3182, and 3364; polychlorinated dibenzo-*p*-dioxins and furans, dioxin-like PCBs, and polybrominated diphenyl ethers (PBDEs) using Methods 3418 and 3430; and pharmaceuticals and personal care products (PPCPs), nonylphenols (NPs), and per- and polyfluoroalkyl substances (PFAS) using Methods 3454, 3550, and 3457, respectively, and mercury (Method 3060) (Table S2).

Wastewater samples were analyzed for polycyclic aromatic hydrocarbons (PAHs), polychlorinated biphenyls (PCBs), and metals, metalloid, and non-metal elements arsenic and selenium using Methods 3265, 3400, 3094, and 3302, respectively (Table S3). PAHs and PCBs were analyzed in surface waters using Methods 3480 and 3459. Lastly, metals, metalloid, and non-metal elements arsenic and selenium were analyzed using Methods 3474 and 3089 (Table S4).

For Methods E3418, E3430, and E3459, Quanlynx Software calculates the Limit of Detection (LOD), which is used as a guide in reporting method detection limits on a per sample basis, taking into consideration matrix effects, sample size, and surrogate recovery.

### Statistical analyses

#### Nested permutation ANOVA

For all analyses (% DNA in tail, phagocytosis assay), we used a permutation 3-way nested General Linear Model (GLM), which is nonparametric and relies on few assumptions about the data (McDonald 2014). For the comet assay, we calculated a test statistic by calculating an F-statistic on the DNA damage for the nested factors mussels and dilutions and the main effect of sampling sites. We then created 10,000 permutations of the data by randomly subsampling with replacement the original observations among and within both nested factors and main effects and computed the value of the F-test statistics for each GLM. We calculated the *p* value by computing the proportion of the permuted distribution of *F*-values greater than or equal to the test statistic computed for the observed data. A *p* value equal to or smaller than 0.05 was considered significant. Mann–Whitney post hoc tests were performed to identify concentrations that differed significantly from the solvent control (0.1% EtOH).

#### Principal component analysis

To assess whether samples contained specific chemical patterns specific to sample type (e.g., influent, effluent, and surface water), the latter was examined by principal components analysis (PCA), using varimax-normalized rotation on untransformed PPCP concentrations. We excluded variables from the PCA that were highly similar to each other, as using all variables resulted in an ill-conditioned matrix, where small changes in the independent variables result in large changes in dependent variables, thus rendering the analysis less reliable. The sample sizes were too low to generate replacement values using maximum likelihood; consequently, we substituted values randomly selected from a uniform distribution between zero and the method detection limit (MDL) for each observation below MDL. Different methods for generating replacement values were inconsequential to the interpretation of the PCA (Figure S2). Statistics were analyzed using Statistica 7 (Statsoft 2004) and Excel (Microsoft 2016).

## Results

### Viability and cell density

#### Freshwater mussel hemocyte assay

Hemocyte density (viable cell count) at the beginning of the experiment ranged from 7·10^5^ to 1·10^6^ cells/mL. After four hours, the hemocyte density was 1.5·10^5^–2.0·10^5^ cells/mL (Table S5). Average hemocyte viability was 94–98% at the beginning of the exposure and 75–79% after 4 h (Table S5), which could be reflected by the adherence of viable cells to the microplate walls, decreasing the proportion of viable cells in suspension. For each exposure, there were no significant differences in cell viability or density compared to the solvent control (*p* ≥ 0.94), indicating that exposure to the surface water and wastewater extracts did not cause significant mortality and was therefore likely not responsible for the decrease in cell viability or density.

#### Rainbow trout leukocyte assays

After exposure, leukocyte viability in controls and solvent controls ranged from 70 to 90% without any significant difference between control and solvent control (Figure S3A). The mean leukocyte viability from control (82 ± 8%) and solvent control (84 ± 9%) treatments was calculated to identify exposure-related changes in viability. No cytotoxic effects were detected at any extract concentration (Figure S3B and C).

### Genotoxicity

**Fig. 2:**
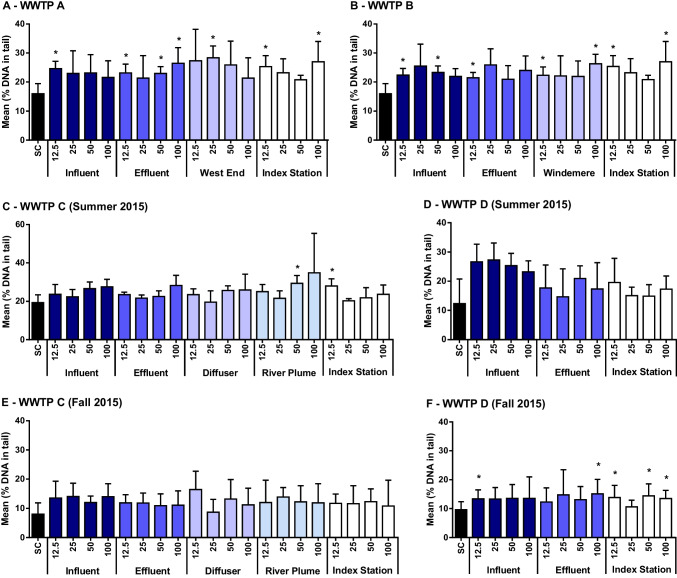
DNA damage in freshwater mussel (*Eurynia dilatata*) hemocytes (mean ± SD, *n* = 4–8; expressed as % DNA in tail) after exposure to increasing concentrations (12.5, 25, 50, and 100%) of extracts of environmental samples from wastewater treatment plant (WWTP) A (A), WWTP B (B), WWTP C summer (C), WWTP D summer (D), WWTP C fall (E), and WWTP D fall (F) and surface water. Asterisks (*) indicate treatments that were significantly different (*p* ≤ 0.05, Mann–Whitney test) from the solvent control (SC)

The influent, effluent, and surface water extracts induced a significant increase in DNA damage, as measured with the Comet assay, in 19 out of 72 samples (Fig. 2). Most of the extracts inducing DNA damage were from the Hamilton Harbour AOC (12 out of 28 samples, Fig. 2A and B). In all cases except for WWTP C in the fall (where no significant induction of DNA damage was observed), the 3-way GLM indicated that sample type (i.e., solvent control, influent, effluent, or surface water) significantly affected DNA damage relative to the solvent control.

In Hamilton Harbour AOC, DNA damage was induced in all three types of samples (influent, effluent, surface water), but did not have a concentration-dependent response. The average % DNA in tail of the solvent control was 16.8%, while most environmental extracts had a mean of 23.5 ± 1.9% DNA in tail, resulting in an average relative increase in DNA damage of ~ 40%. In contrast, surface water extracts from Humber Bay induced a significant increase in DNA damage in two samples collected in the summer, which were not related to WWTP C: from the river plume and the index station (Fig. 2C). Influent and effluent extracts did not induce any significant increase in % DNA in tail in either season (Fig. 2C and E). Lastly, five of the 12 extracts from Toronto Harbour induced significantly greater % DNA in tail than the solvent control in the fall (Fig. 2F): one sample from the influent and effluent and three samples from the index station. Although samples from WWTP D influent had, on average, more than a doubling of the % DNA in tail compared to the solvent control, these were not significantly different due to the high variance of the solvent control (Fig. 2D).

Overall, although we observed increased DNA damage in mussels exposed to environmental samples relative to those in the solvent control, there was no clear pattern of DNA damage among influent, effluent, and surface water samples within each region. Lastly, the % DNA in tail varied significantly among individual mussels for each of the locations and sampling seasons for the comet assay (3-way nested GLM; *p* = 0.000 for all comparisons), indicating that the responses to effluent or the efficacy of the assay varied among individuals.

### Immunotoxicity

**Fig. 3:**
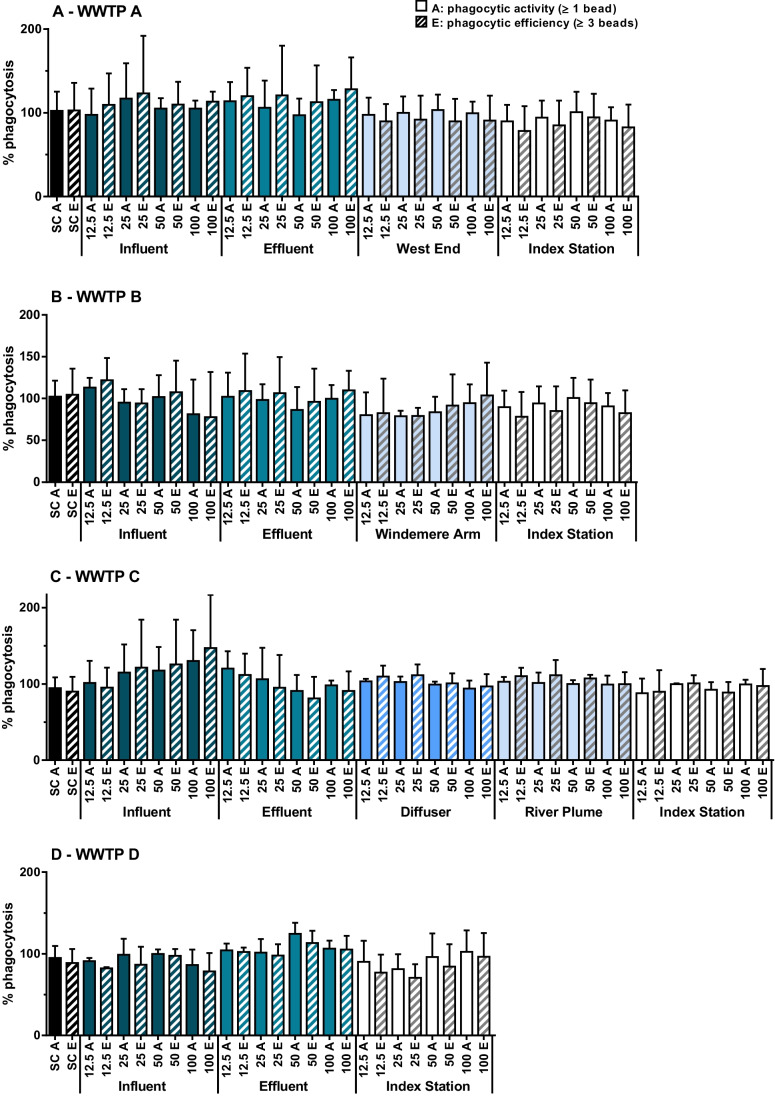
Phagocytosis in rainbow trout (*Oncorhynchus mykiss*) leukocytes (mean ± SD, *n* = 3; expressed as % relative phagocytosis) after exposure to increasing concentrations (12.5, 25, 50, and 100%) of extracts of environmental samples from wastewater treatment plant (WWTP) A (A), WWTP B (B), WWTP C summer (C), and WWTP D summer (D) and surface water. Solid bars represent mean % activity (≥ 1 bead), and hatched bars represent mean % efficiency (≥ 3 beads). SC, solvent control

Phagocytic efficiency (cells having engulfed ≥ 3 beads) was significantly affected by sample type (i.e., influent, effluent, or surface water) in WWTP A (*p* < 0.001), WWTP B (*p* = 0.036), and WWTP C (*p* = 0.020) sample extracts (Fig. 3). The dilution of the extract (12.5, 25, 50, or 100% of initial sample) did not significantly affect phagocytic efficiency or activity in trout leukocytes. A significant difference in phagocytic efficiency was only observed in fish exposed to extracts from WWTP A compared to the solvent control (*p* = 0.01); however, the post hoc test could not identify any specific treatment that differed from solvent control.

Phagocytic activity (cells having engulfed ≥ 1 bead) was significantly affected by sample type only in Humber Bay extracts (*p* = 0.024). No effect on phagocytosis (activity or efficiency) was observed in any of the Toronto Harbour extracts. There were no significant differences in any sample extract concentration and their corresponding solvent control for any of the sites (Fig. 3).

### Chemical analysis

The measured concentrations of chemicals and parameters discussed below are presented in Table 2 (Hamilton Harbour AOC) and Table 3 (Toronto and Region AOC). A complete list of PPCPs, PCBs, dioxins and furans, nonylphenols, metals, and conventional water quality measures in influent, effluent, and surface waters are presented in Table S6 (Hamilton Harbour AOC) and Table S7 (Toronto and Region AOC). Given the large scale of the study, a single replicate sample was collected at each location, site, and season, with emphasis on matching influent, effluent, and surface water samples (duplicate samples were collected for QA/QC, for which the mean value of the sample is reported). Data on all compounds were not analyzed for every sampling time point. Concentrations of PPCPs in influent from WWTP C and WWTP D were generally greater or similar to those in their corresponding effluent, with the exception of carbamazepine, lidocaine, sulfamethoxazole, and trimethoprim, for which concentrations increased in effluent, reflecting poor removal from wastewater and/or concentrating effect. We could not make a similar comparison with WWTP A and WWTP B, because the influent samples were not submitted for chemical analysis. However, in all locations, concentrations of PPCPs in surface water samples were lower than in comparison to those in the corresponding influent and/or effluent. For PCBs, patterns differed between AOCs: concentrations were independent of treatment in Hamilton Harbour AOC (concentrations in surface water were not necessarily lower than those in the effluent (concentrations in influent were not measured)), whereas in the Toronto and Region AOC, PCB concentrations decreased with treatment and compared to concentrations in surface water. Concentrations of organic compounds (nonylphenols, dioxins, and furans) also decreased with sewage treatment and were lower in surface water samples. Ammonia/ammonium concentrations decreased following treatment of the influent, but nitrite and nitrate concentrations increased in effluents.

**Table 2:** Concentration of compounds and nutrients detected in the Hamilton Harbour Area of Concern, including wastewater treatment plant (WWTP) A and B, and surrounding surface waters

	Site/WWTP	WWTP A		WWTP B		West End Index Station		Hamilton Harbour Index Station		Windermere Arm Index Station	
	Sample type	Effluent		Effluent		Surface		Surface		Surface	
	Date	Nov-14		Nov-14		Jul-14		Jul-14		Jul-14	
PPCPs (ng/L)	Bezafibrate	107.3		34.3		2.0	<	2.0	<	2.0	<
	Caffeine	360.0		187.0		10.0	<	10.0	<	10.0	<
	Carbamazepine	306.3		277.3		43.0		42.0		49.0	
	Diclofenac	830.0		517.0		5.0	<	5.0	<	5.0	<
	Lidocaine	183.0		138.3		13.0		11.0		17.0	
	Triclocarban	6.0		4.7		0.8		2.3		1.1	
	Trimethoprim	239.3		143.0		13.0		18.0		14.0	
PCBs (pg/L)	PCB105	3.70	<	17.33	#	5.20	<	9.20	<	17	<
	PCB118	12.33	<	52.00	#	18.00	<	27.00	<	60	<
NPs (ng/L)	4-Nonylphenol	80.3	*	46.7	*	28.0		24.0		20.0	*
Dioxins/furans (pg/L)	Octachlorodioxin	0.76	<	1.7	<	4.5		3.5		4.1	
Nutrients (mg/L)	Ammonia (NH_3_) + ammonium (NH_4_ +)	0.055		0.127		0.083		0.089		0.140	
	Nitrate (NO_3_-) + nitrite (NO_2_-)	5.981		15.967		2.350		2.560		2.710	
TSD (mg/L)	Suspended solids	0.8		4.433		5.4		4.9		5.7	
BOD	Biochemical oxygen demand	1.15		4.67		2.33		8.03		10.27	
Conductivity (µS/cm)	Conductivity	1123		1780		760		774		786	

Annotations: < indicates the method detection limit (MDL) or values measured below the MDL; # indicates values where one of the two replicates was below the MDL; * indicates samples for which recommended holding times were exceeded

**Table 3:** Concentration of compounds and nutrients detected in the Toronto and Region Area of Concern, including wastewater treatment plant (WWTP) C and D and surrounding surface waters

	Site/WWTP	WWTP C								Diffuser		River plume		Humber Bay Index Station		WWTP D								Toronto Harbour Index Station			
	Sample type	Influent		Effluent		Influent		Effluent		Surface		Surface		Surface		Influent		Effluent		Influent		Effluent		Surface		Surface	
	Date	Jun-15		Jun-15		Oct-15		Oct-15		Jun-15		Jun-15		Jun-15		Jun-15		Jun-15		Oct-15		Oct-15		Jun-15		Jul-15	
PPCPs (ng/L)	Bezafibrate	80.4		37.7		101.6		46.3		2	<	2	<	2	<	74.8		23.8		132.8		46.7		2	<	2	<
	Caffeine	22,800		269.3		5040		400		150.0		44		33		456.0		166.3		2556		63.5		79		120	
	Carbamazepine	940		370.0		255.2		501.2		22.0		3.8		3.5		84.0		205.2		210.8		359.7		4.2		5.4	
	Diclofenac	944.0		709.3		688.0		547.5		40.0		5	<	5	<	424.0		273.3		520.0		506.7		5	<	5	<
	Lidocaine	314.8		419.7		162.4		384.7		19.0		2.0		2.3		145.2		211.5		147.6		271.2		2.2		2.5	
	Triclocarban	188.4		8.5		191.2		48.5		1.4		0.5	<	0.5	<	60.4		13.6		132.0		26.3		0.5	<	0.5	<
	Trimethoprim	133.6		132.7		90.4		147.3		2.1		2.0	<	2	<	50.0		109.4		108.4		176.3		2.0	<	2.0	<
PCBs (pg/L)	PCB105	240.0		40.0		680		38	<	7.4	<	3.1	<	6.6	<	190.0		11.00	<	110.0		15.00	<	5.30	<	3.95	<
	PCB118	620.0		120.0		1600		110	<	18.0	<	8.1	<	26.0	<	500.0		32.0	<	290.0		47.0	<	16.0	<	12.0	<
NPs (ng/L)	4-Nonylphenol	4100		85.3		2300		47.7		22	*	20	*	20	*	5100		94		4000		270.0		20	*	28	*
Dioxins and furans (pg/L)	Octachlorodioxin	42		1.8	<	30		1.9		7.4		2.1	<	2.2		28		2.3		200		1.2	<	1.8		1.075	<
Nutrients (mg/L)	Ammonia (NH_3_) + ammonium (NH_4_ +)	13.4		0.101		27.1		0.312		0.093		0.058		0.044		18.0		0.465		27.6		6.707		0.058		0.041	
	Nitrate (NO_3_-) + nitrite (NO_2_-)	0.02	<	7.720		0.02	<	16.63		1.31		0.319		0.291		0.061		14.667		0.02	<	9.277		0.316		0.275	
TSD (mg/L)	Suspended solids	171		5.67		84.1		8.47		21.9		4.6		1.3	<	317		11.1		162		4.13		1.3	<	1.3	<
BOD	Biochemical oxygen demand	2.7		3.1		1.2		1.6		1.4		1.1		1.6		6.27		295		265		346		205		3.4	
Conductivity (µS/cm)	Conductivity	1370		1240		1230		1047		497		332		319		1250		1170		1020		915		348		365	

Annotations: < indicates the method detection limit (MDL) or values measured below the MDL; * indicates samples for which recommended holding times were exceeded

### Principal component analysis (PCA)

**Fig. 4:**
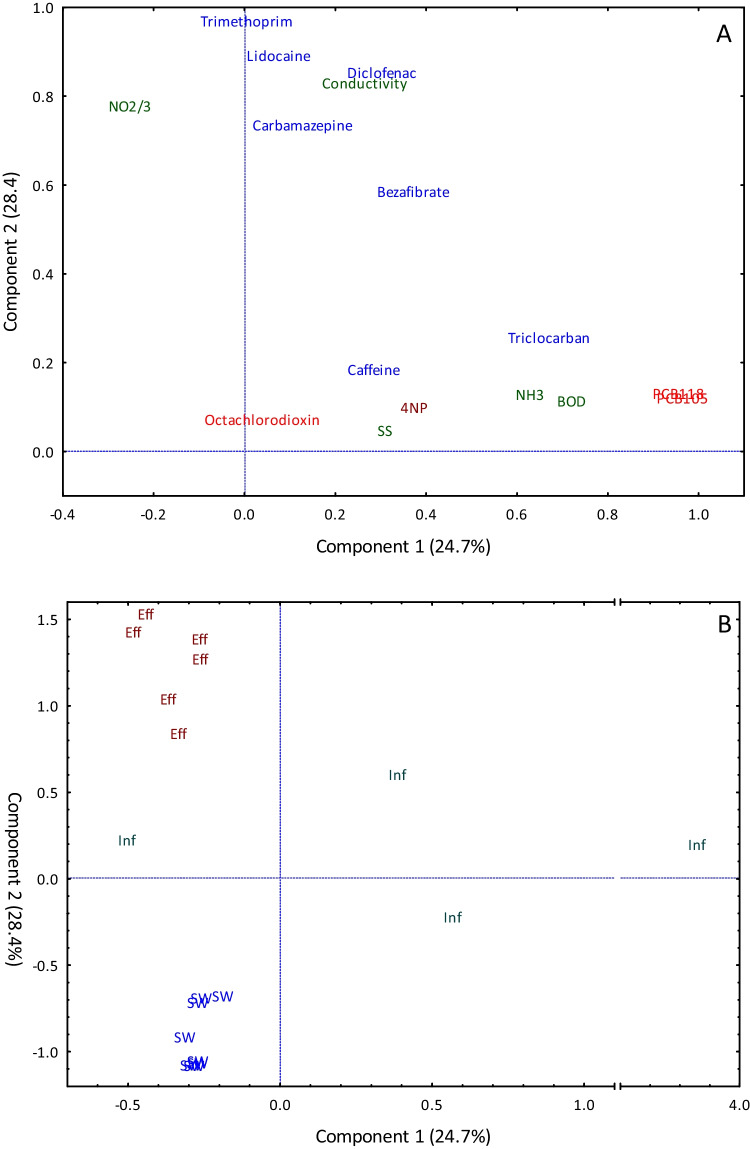
Principal component loadings (A) and by sample type (B) for chemicals measured in influent, effluent, and surface waters with pharmaceutical and personal care products (PPCPs) (blue), Polychlorinated biphenyls (PCBs) dioxins (red), nonylphenols (brown), and nutrients, conductivity and biological oxygen demand (dark green). Note the scale breaks on the *x*-axis. NO_2/3_, Nitrite + nitrate; ammonia/ammonium; SS, suspended solids; 4NP, 4 nonylphenol, NH_3_, nitrate; BOD, biological oxygen demand; Inf, influent; Eff, effluent; SW, surface water

Of the top 4 principal components extracted, 90.8% of the total variance was explained after varimax normalized rotation, the first component explaining 24.7% of the variance, and the second component, 28.4%. Principal Component 1 (PC1) had large positive loadings (> 0.7) with non-ortho PCBs, NH_3_, and BOD (Fig. 4A), and PC2 had large positive loadings (> 0.7) with four PPCPs (carbamazepine, diclofenac, lidocaine, and trimethoprim, corresponding to those compounds that did not decrease with sewage treatment) and with nitrate/nitrite and conductivity. The PCA separated the four influent samples on the first component, while the second component separated the samples by type (Fig. 4B). Effluent samples were separated from other sample types based on the positive loadings on PC2, whereas the surface water samples were separated based on negative loadings on the PC2, associated with the reduced concentration of those four PPCPs in surface water compared to influent and effluent. Influent samples generally had relatively elevated concentrations of nutrients, particularly NH_3_, which, as expected, was reduced in effluent and surface water relative to influent. The fall influent sample from was separated from all other samples on the PC1, due to elevated concentrations of PCBs and nonylphenols (Fig. 4B). Conversely, the fall influent sample from WWTP D did not separate from the other samples on the PC1, due to lower concentrations of PCBs, and an exceptionally elevated concentration of octachlorodioxin and BOD (Fig. 4B). The PCA did not discriminate among groups when the samples were categorized by location (data not shown), indicating that sample type (influent, effluent, and surface water) played a greater role in determining chemical composition than sample location (Hamilton Harbour, Humber Bay, Toronto Harbour) or season (spring or fall).

## Discussion

Municipal wastewater is one of the largest sources of surface water pollution in Canada and across the world. In 2017, 5.91 million liters of water were released into Canadian surface waters, 95.6% of which had received at least primary treatment (Statistics Canada 2019; Environment and Climate Change Canada 2020). Municipal effluents can impair the immune system of fish and bivalves (Hébert et al. 2008; Muttray et al. 2012; Gagné et al. 2012, 2013) and induce genotoxicity in exposed aquatic animals (Lah et al. 2004). The Neptune European program (www.eu-neptune.org) recommends the use of in vitro and in vivo bioassays for the management of municipal effluents, to address the complexity of assessing the toxicity of effluent mixtures.

The objective of the present study was to assess the genotoxic and immunotoxic potential of untreated wastewater influent, treated wastewater effluent, and surface water from two areas impacted by anthropogenic activities, and evaluate the usefulness of these in vitro assays as screening tools for the identification of potentially harmful WWE and surface waters. By collecting samples in the summer and the fall (Toronto and Region AOC), we hoped to gain some insight into seasonal differences in the influence of the watershed during periods of high flow and low flow. Our expectation was that in areas where municipal wastewater effluents contributed significantly to contamination of the aquatic system, (treated) WWTP effluents would be less toxic relative to the (untreated) influent, and surface waters that receive wastewater effluents would be less toxic than both influents and effluents. We also identified and quantified numerous chemicals present in the environmental sample extracts, to characterize the sources of contamination.

Given the significant extent and levels of pollution in the Hamilton Harbour AOC, we expected—and found—observable genotoxicity in extracts from every type of sample collected. Surface water samples from West End, Windermere Arm, and Hamilton Harbour Index Station showed a significant increase in DNA strand breaks in freshwater mussel hemocytes (Fig. 2). Surface waters and sediments from Hamilton Harbour AOC have been reported to induce genotoxicity, thought to be due to past and present industrial activities: surveys of Brown Bullheads (*Ameiurus nebulosus*) indicated that wild fish from this AOC had a significantly higher occurrence of tumors and other deformities compared to other AOCs (Baumann 2010), and sediments from Hamilton Harbour induced liver tumors in rainbow trout (Metcalfe et al. 1988). There appears to be little information available on genotoxic effects in other biota. Some influent and effluent extracts from WWTP A and WWTP B also induced a significant increase in % DNA in tail in the Comet assay, suggesting that WWTPs may also be contributing to the observed genotoxicity.

In Toronto and Region AOC, we detected significant differences in % DNA in tail in samples collected from the Humber River plume and the index station in the summer, but did not detect any differences in DNA damage in extracts from WWTP C (summer), or in any of the sample extracts from the fall. These results suggest the Humber River could be a source of genotoxic compounds that did not originate from the WWTP; the absence of DNA damage from sample extracts from the fall could indicate seasonal variations. Unfortunately, chemical analysis was only completed on the surface water samples collected in the summer, so we were unable to compare samples in light of chemicals detected. Based on the results of the chemical analysis and the PCA, samples did not appear to contain any of the measured contaminants in greater concentrations than the influent or effluent. In Toronto Harbour, despite large increases in DNA damage in influent extracts collected from WWTP D in the summer (more than doubling of DNA damage), large variability prevented the identification of significant differences. In contrast, in extracts from samples collected in the fall, significant DNA damage was identified in one influent and effluent sample from WWTP D, and in three of the four samples from surface water from Toronto Harbour. There appears to be few studies assessing the presence of genotoxic compounds in the Toronto and Region AOC; fish collected from this AOC did not have significantly greater tumors compared to baseline data for the Great Lakes (Baumann 2010; Environment Canada 2011a).

In the present study, we did not detect any consistent decrease in the genotoxicity of influent vs. effluent extracts, or with dilution of extracts, despite the observed differences in chemical composition among sample type. It is possible that the eightfold difference in exposure from the highest concentration to the 12.5% exposure was not sufficient to reduce the genotoxicity in a concentration-dependent manner. Exposure to contaminants can also alter DNA repair mechanisms, for example, certain heavy metals, PAHs, or estrogenic compounds (reviewed in Kienzler et al. 2013). In a similar study, although acute toxicity of three WWTP effluents to zebrafish (*Danio rerio*) was dramatically reduced following treatment, genotoxicity increased (Zhang et al. 2013). Langevin et al. (1992) found that despite finding site differences in the genotoxicity of water and sediment samples throughout the St. Lawrence River (Canada), genotoxicity did not correlate with measured concentrations of any mutagens or other toxic chemicals. Further treatment of wastewater may be required to remove the chemical elements causing genotoxicity despite improvements to indicators of acute toxicity.

There were no significant differences in phagocytic efficiency of rainbow trout kidney leukocytes between individual extract concentrations and the respective solvent controls, but we observed significant differences between sample types in all but the Toronto summer extracts (Fig. 3A-C). The effect of municipal effluents on phagocytosis in fish has been previously assessed (Gagné et al. 2013). Rainbow trout leukocytes exposed to extracts of municipal influents and effluents in vitro for 24 h showed changes (increases as well as decreases) in phagocytic activity (Gagné et al. 2013), although the differences in phagocytic activity were heavily influenced by leukocyte viability in the most concentrated wastewater. Phagocytosis was reported to be a good immunological biomarker for heavy metal exposure, to which fish were particularly sensitive (Fournier et al. 2000). Studies reported that pharmaceuticals had the capacity to increase (bezafibrate, gemfibrozil, and trimethoprim) or decrease (novobiocin, morphine, trimethoprim, and erythromycin) phagocytosis in *Elliptio sp.* (Gagné et al. 2006) or *Mytilus sp.* (Lacaze et al. 2015). In our study, pharmaceuticals were some of the most frequently-detected compounds (Tables 2 and 3). Municipal wastewater effluents are generally the main sources for pharmaceuticals in surface waters. Some of the compounds reported to increase phagocytosis, bezafibrate, gemfibrozil, trimethoprim, erythromycin (albeit with threshold effects at in vitro concentrations of 2–3 μM (Gagné et al. 2006) or 15 mg/L (Lacaze et al. 2015)) were detected at ng/L concentrations in the extracts WWTP A, WWTP B, WWTP C, and WWTP D influent and effluent, as well as Hamilton Harbour surface water (only trimethoprim) (Tables 2 and 3).

The PCA could readily separate samples by type (e.g., influent, effluent, and surface waters), based on their chemical composition. Influent had the largest variability in chemical composition, but generally had the highest concentrations of PCBs and BOD, as well as elevated concentrations of PPCPs. Effluent had either similar or occasionally greater concentrations of PPCPs (diclofenac, trimethoprim, and carbamazepine), in contrast with PCBs and other highly lipophilic compounds that tend to sorb to organics, and tend to be better removed during wastewater treatment (Vogelsang et al. 2006). Conversely, although many PPCPs are degraded or otherwise removed during wastewater treatment, concentrations of some PPCPs can remain elevated, or may under certain conditions increase from influent to effluent (e.g., diclofenac and carbamazepine (Kasprzyk-Hordern et al. 2009)). In the present study, trimethoprim, lidocaine, and carbamazepine increased through sewage treatment, while diclofenac and bezafibrate were poorly removed, as best illustrated by the PC2 of the PCA, which separated samples according to type (Fig. 4A). While influents were characterized by elevated ammonia concentrations, effluents had elevated nitrite/nitrate concentrations, suggesting incomplete denitrification of the nitrate and nitrite byproduct of the initial nitrification of ammonia (Du et al. 2019). The PC1 of the PCA further separated influent extracts, while the summer samples from both Humber Bay and Toronto Harbour were distributed along the center of the graph, the fall samples were further separated due to, notably, elevated octachlorodioxin and BOD (Toronto Harbour) and PCB 105, PCB 118, and nonylphenol (Humber Bay). Further research would be needed to identify contamination sources and verify whether these differences are driven by seasonal variations.

Wastewater effluents can have dramatic effects on aquatic organisms downstream of the outflow; Gillis et al. (2017a) examined wild mussel populations surrounding a large secondary treatment plant (> 200,000 serviced) and reported that poor water quality in the receiving environment likely contributed to the observed mussel extirpation zone downstream of the outflow. Thus, upgrades to WWTPs can result in substantial improvements in effluent quality. For example, following upgrades to the WWTP in Kitchener (ON, Canada) to include nitrifying activated sludge treatment (at a cost of approximately $350 M), the percent of intersex male darters (*Etheostoma caeruleum*) downstream of the outflow dropped from 70 to 100% prior to the upgrade, to < 10% following the upgrade (Hicks et al. 2017). Mussels have been reported to accumulate many pharmaceuticals (Gilroy et al. 2014, 2017; de Solla et al. 2016), and there is a need to assess the toxicity of pharmaceuticals to freshwater organisms, both individually and in mixtures. Monitoring surface waters receiving WWE is needed to assess the effectiveness of WWTP upgrades to improve water quality, especially given the costs of such infrastructure investments. For example, WWTP B is currently undergoing infrastructure upgrades to tertiary treatment (City of Hamilton 2022), which is expected to improve the quality of the effluent and, hence, mitigate toxicity to aquatic life in the receiving environment.

## Summary and conclusions

Water extracts from municipal wastewater treatment plant influent and effluent and surface water from three locations along Lake Ontario induced DNA damage at low amplitude and frequencies in the hemocytes of the freshwater mussel *Elliptio dilatata*, after a 4-h in vitro exposure. The genotoxicity of samples of all types from Hamilton Harbour AOC was elevated compared to the solvent control. Considering that the harbor is known for its sediments contaminated with genotoxic compounds, we suspected that local contamination of the receiving water may be contributing to the observed genotoxicity. Extracts from the Toronto and Region AOC had fewer incidences of increased DNA damage relative to the solvent controls; the DNA damage observed in Humber Bay extracts did not appear to be related to WWTP C, while DNA damage observed in Toronto Harbour was of mixed sources (influent and index station). Greater replication and longer exposure times would increase sensitivity to indirect genotoxicants, while serial dilutions encompassing a greater range of concentrations (e.g., using a logarithmic or semi-logarithmic scale) may improve our ability to observe a concentration response. 

We observed significant differences between sample types in the phagocytic activity of rainbow trout kidney leukocytes (in all but one sample set); however, no differences were observed between individual extract concentrations and the respective solvent controls. The risks and hazards of municipal wastewater effluents have frequently been estimated using both traditional chemical and biochemical measures of wastewater composition (Metcalfe et al. 2013; Kiesling et al. 2019; Kleywegt et al. 2019) and by toxicity testing using whole organisms such as fish and mollusks (Zhang et al. 2013; Gillis et al. 2014). Nonetheless, in vitro and in vivo bioassays, such as induction of genotoxicity, may detect impairment to aquatic animals that are not readily observed through alterations of higher-level function. These assays are cost-effective in comparison with multi-contaminant analyses, and thus, their development and validation as rapid screening tools will contribute to the improvement of ecotoxicological assessments of mixtures.

## Supplementary Information

Below is the link to the electronic supplementary material.

Supplementary file1 (PDF 1783 KB)

## Data Availability

Data and materials are available upon request. Email: eve.gilroy@ec.gc.ca.
