# Supplementary material for: In vitro assessment of the genotoxicity and immunotoxicity of treated and untreated municipal effluents and receiving waters in freshwater organisms
Source: Environ Sci Pollut Res Int. 2023 Apr 15;30(23):64094–110. doi: 10.1007/s11356-023-26845-1 (PMC10172253; doi:10.1007/s11356-023-26845-1)
Supplement: Supplementary file 1 — Supplementary file1 (PDF 1783 KB) [file 11356_2023_26845_MOESM1_ESM.pdf]

Supplementary Information

In vitro assessment of the genotoxicity and immunotoxicity of treated and untreated municipal effluents and receiving waters in freshwater organisms

Ève AM Gilroy[1,2], Christine Kleinert[3,4], Émilie Lacaze[4,5], Sheena D Campbell[2], Sara Verbaan[2,6], Chantale André[5], Kara Chan[1], Patricia L. Gillis[1], Joel Klinck[6], François Gagné[5], Michel Fournier[4], Shane R de Solla[3]

[1] Aquatic Contaminants Research Division, Environment and Climate Change Canada, Burlington, ON
[2] Green House Science, Burlington, ON
[3] Ecotoxicology and Wildlife Health Division, Environment and Climate Change Canada, Burlington, ON
[4] Institut National de la Recherche Scientifique-Centre Armand-Frappier Santé Biotechnologie, Laval, QC
[5] Aquatic Contaminants Research Division, Environment and Climate Change Canada, Montréal, QC
[6] Redeemer University, Ancaster, ON

Table S1: Details on the number of samples collected by the Ontario Ministry of the Environment, Conservation and Parks for the assessment of the immunotoxicity and genotoxicity treated and untreated municipal effluents and surface waters in two Canadian Areas of Concern in Lake Ontario, ON, Canada.

Species	Assay	Sample Location	WWTP	# of sample extracts	# of collections (dates)	# of controls (EtOH, H_2O_2)	# of treatments (dilutions)	# of replicates (hemolymph or leucocytes)	Total number of samples
Freshwater mussels	genotoxicity	Hamilton Harbour	A & B	7	1	2	4	4	144
		Humber Bay	C	5	2	2	4	8	448
		Toronto Harbour	D	3	2	2	4	8	320
Rainbow trout	immunotoxicity	Hamilton Harbour	A & B	7	1	1	4	3	96
		Humber Bay	C	5	1	1	4	3	72
		Toronto Harbour	D	3	1	1	4	3	48

Table S2. List of analytes, their methods, and method detection limits for Surface Water and Wastewater Samples.

Parameter name	Practical Quantitation Limit	Units
MECP Method 3182 – "THE DETERMINATION OF BIOLOGICAL OXYGEN DEMAND IN SURFACE WATERS AND SEWAGE EFFLUENTS BY DISSOLVED OXYGEN METER" (500 ml PET)		
MECP Method 3188 – "THE DETERMINATION OF SOLIDS IN LIQUID MATRICES BY GRAVIMETRY" (500 ml PET)		
MECP Method 3218 – "THE DETERMINATION OF CONDUCTIVITY, pH AND ALKALINITY INWATER AND EFFLUENTS BY POTENTIALITY" (500 ml PET)		
MECP Method 3364 – "THE DETEMRINATION OF AMMONIANITROGEN, NITRITE NITROGEN, NITRITE PLUS NITRATE NITROGEN AND REACTIVE ORTHO-PHOSPHATE IN SURFACE WATER, DRINKING WATER AND PRECIPITATION BY COLOURIMETRY" (500 ml PET)		
BOD	2	mg/L
Suspended solids	1	mg/L
Total Kjeldahl Nitrogen	0.04	mg/L
Total Phosphorus as P	0.04	mg/L
Conductivity	5	uS/cm
pH	0.1	none
Alkalinity; total fixed endpt	2.5	mg/L CaCO3
Nitrogen; ammonia+ammonium	0.1	mg/L
Nitrogen; nitrite	0.005	mg/L
Nitrogen; nitrate+nitrite	0.025	mg/L
Phosphorus; phosphate	0.0025	mg/L
MECP Method 3418 "THE DETERMINATION OF POLYCHLORINATED DIBENZO-P-DIOXINS, POLYCHOLRDIBENZOFURANS AND DIOXIN-LIKE POLYCHLORINATED BIPHENYLS IN ENVIRONMENTAL SAMPLES BY GAS CHROMATOGRAPHY-MASS SPECTROMETRY (GC-MS) (1L glass amber jar)		
Octachlorofuran	Quanlynx Software calculates the Limit of Detection (LOD), which is used as a guide in reporting method detection limits on a per sample basis, taking into consideration matrix effects, sample size, and surrogate recovery.	pg/L
Octachlorodioxin		pg/L
2378-tetrachlorofuran		pg/L
2378-tetrachlorodioxin		pg/L
12378-pentachlorofuran		pg/L
23478-pentachlorofuran		pg/L
12378-pentachlorodioxin		pg/L
123478-hexachlorofuran		pg/L
123678-hexachlorofuran		pg/L
234678-hexachlorofuran		pg/L
123789-hexachlorofuran		pg/L
123478-hexachlorodioxin		pg/L
123678-hexachlorodioxin		pg/L
123789-hexachlorodioxin		pg/L
1234678-heptachlorofuran		pg/L
1234789-heptachlorofuran		pg/L
1234678-heptachlorodioxin		pg/L
TEQ NATO 1988 DIOX (ND-0)		pg/L
TEQ NATO 1988 DIOX (ND-1)		pg/L
TEQ NATO 1988 DIOX (ND-1/2)		pg/L
TEQ WHO 2005 DIOX (ND-0)		pg/L
TEQ WHO 2005 DIOX (ND-1)		pg/L
TEQ WHO 2005 DIOX (ND-1/2)		pg/L

MECP Method DLPCB 3418 "THE DETERMINATION OF POLYCHLORINATED DIBENZO-P-DIOXINS, POLYCHOLRDIBENZOFURANS AND DIOXIN-LIKE POLYCHLORINATED BIPHENYLS IN ENVIRONMENTAL SAMPLES BY GAS CHROMATOGRAPHY-MASS SPECTROMETRY (GC-MS)" (I L glass amber jar)

3,4,4',5-tetrachlorobiphenyl		pg/L
3,3',4,4'-tetrachlorobiphenyl		pg/L
2'3,4,4',5-pentachlorobiphenyl		pg/L
2,3'4,4',5-pentachlorobiphenyl		pg/L
2,3,4,4',5-pentachlorobiphenyl		pg/L
2,3,3'4,4'-pentachlorobiphenyl		pg/L
3,3'4,4',5-pentachlorobiphenyl		pg/L
23',44',55'-hexachlorobiphenyl		pg/L
2,3,3'4,4'5-hexachlorobiphenyl		pg/L
2,3,3'44'5'-hexachlorobiphenyl		pg/L
3,3'4,4'55'-hexachlorobiphenyl		pg/L
233'44'55'-heptachlorobiphenyl		pg/L
TEQ WHO 2005 DLPCB (ND-0)		pg/L
TEQ WHO 2005 DLPCB (ND-1/2)		pg/L
TEQ WHO 2005 DLPCB (ND-1)		pg/L

MECP Method 3430 "THE DETERMINATION OF BROMINATED FLAME RETARDANTS (BFRS) IN ENVIRONMENTAL MATRICES BY GAS CHROMATOGRAPHY-HIGH RESOLUTION MASS SPECTROMETRY (GC-HRMS)" (1L glass amber jar)

BDE-17	Quanlynx Software calculates the Limit of Detection (LOD), which is used as a guide in reporting method detection limits on a per sample basis, taking into consideration matrix effects, sample size, and surrogate recovery.	ng/L
BDE-28		ng/L
BDE-49		ng/L
BDE-71		ng/L
BDE-47		ng/L
BDE-66		ng/L
BDE-77		ng/L
BDE-100		ng/L
BDE-119		ng/L
BDE-99		ng/L
BDE-85		ng/L
BDE-126		ng/L
BDE-154		ng/L
BDE-153		ng/L
BDE-139		ng/L
BDE-140		ng/L
BDE-138		ng/L
BDE-156/169		ng/L
BDE-184		ng/L
BDE-183		ng/L
BDE-191		ng/L
BDE-180		ng/L
BDE-171		ng/L
BDE-201		ng/L
BDE-204		ng/L
BDE-197		ng/L
BDE-203		ng/L
BDE-196		ng/L
BDE-205		ng/L
BDE-208		ng/L
BDE-207		ng/L
BDE-206		ng/L
BDE-209		ng/L

MECP Method E3550 “THE DETERMINATION OF NONYLPHEMOL AND NONYLPHENOL ETHOXYLATES IN WATER BY LIQUID CHROMATORGRAPHY TANDEM MASS SPECTROMETRY (LC-MS/MS)” (1L glass amber jar)

4-n-Nonylphenol Diethoxylate	5	ng/L
4-Nonylphenol	5	ng/L
4-Nonylphenol Monoethoxylate	20	ng/L
4-Nonylphenol Diethoxylate	5	ng/L

Pharmaceuticals and personal care products
MECP method E3454 – “ THE DETERMINATION OF PHARMACEUTICALS AND PERSONAL CARE PRODUCTS IN (PPCPs) ENVIRONMENTAL MATRICES BY LC-MS-MS ANALYSIS” (1L glass amber jar)

Acetaminophen (paracetamol)	10	ng/L
alpha-Estradiol	500	ng/L
Atenolol	50	ng/L
Atorvastatin	1	ng/L
beta-Estradiol	500	ng/L
Bezafibrate	2	ng/L
Bisphenol A	20	ng/L
Caffeine	10	ng/L
Carbadox	20	ng/L
Carbamazepine	0.5	ng/L
Chloramphenicol	5	ng/L
Chlortetracycline	20	ng/L
Ciprofloxacin	5	ng/L
Clofibric Acid	2	ng/L
DEET	5	ng/L
Diclofenac	5	ng/L
Diethylstilbestrol	500	ng/L
Doxycycline	5	ng/L
Enrofloxacin	1	ng/L
Equilin	50	ng/L
Erythromycin	1	ng/L
Estriol	100	ng/L
Estrone	2	ng/L
Ethynylestradiol	500	ng/L
Gemfibrozil	2	ng/L
Glipizide	2	ng/L
Glyburide	1	ng/L
Hydrocortisone	20	ng/L
Ibuprofen	20	ng/L
Indomethacin	2	ng/L
Ketoprofen	2	ng/L
Lasalocid A	1	ng/L
Lidocaine	0.5	ng/L
Lincomycin	0.5	ng/L
Meclocycline	2	ng/L
Monensin Sodium	1	ng/L
Naproxen	10	ng/L
Norethindrone	5	ng/L
Norfloxacin	5	ng/L
Oxolinic Acid	1	ng/L
Oxybenzone	5	ng/L
Oxytetracycline	10	ng/L
Progesterone	2	ng/L
Roxithromycin	10	ng/L
Sulfachloropyridazine	2	ng/L
Sulfadiazine	5	ng/L
Sulfadimethoxine	1	ng/L
Sulfamerazine	5	ng/L
Sulfamethazine	2	ng/L

Sulfamethizole	2	ng/L
Sulfamethoxazole	2	ng/L
Sulfathiazole	2	ng/L
Tetracycline	10	ng/L
Triclocarban	0.5	ng/L
Triclosan	20	ng/L
Trimethoprim	2	ng/L
Warfarin	0.5	ng/L

MECP Method E3457 "THE DETERMINATION OF PERFLUORINATED ALKYL ACIDS IN WATERS BY LIQUID CHROMATOGRAPHY / MASS SPECTROMETRY" (500 ml PET)

Perfluorohexane sulfonate	10	ng/L
Perfluorooctane sulfonate	5	ng/L
Perfluorodecane sulfonate	10	ng/L
Perfluorooctane sulfonamide	5	ng/L
Perfluoroheptanoic acid	10	ng/L
Perfluorooctanoic acid	5	ng/L
Perfluorononanoic acid	5	ng/L
Perfluorodecanoic acid	5	ng/L
Perfluoroundecanoic acid	10	ng/L
Perfluorododecanoic acid	10	ng/L

MECP Method 3060 "THE DETERMINATION OF MERCURY IN WATER, LIQUID INDUSTRIAL WASTE AND LANDFILL LEACHATE SAMPLES BY COLD VAPOUR-FLAMLESS ATOMIC ADSORPTION SPECTROPHOTOMETRY (CV-FAAS)"

Mercury	0.02	ug/L

Table S3. List of analytes, their methods, and method detection limits for Wastewater samples

Parameter name	Practical Quantitation Limit	Units
MECP Method E3265 - "THE DETERMINATION OF POLYCLYCLIC AROMATIC HYDROCARBONS IN WATER BY GAS CHROMATOGRAPHY/MASS SPECTROMETRY" (1L glass amber jar)		
1-chloronaphthalene	500	ng/L
1-methylnaphthalene	500	ng/L
2-chloronaphthalene	500	ng/L
2-methylnaphthalene	500	ng/L
5-nitroacenaphthene	500	ng/L
Acenaphthene	500	ng/L
Acenaphthylene	500	ng/L
Anthracene	500	ng/L
Benz(a)anthracene	500	ng/L
Benzo(a)anthracene	500	ng/L
Benzo(a)pyrene	500	ng/L
Benzo(b)fluoranthene	500	ng/L
Benzo(e)pyrene	500	ng/L
Benzo(g,h,i)perylene	500	ng/L
Benzo(k)fluoranthene	500	ng/L
Chrysene	500	ng/L
Dibenzo(a,h)anthracene	500	ng/L
Dibenz(a,h)anthracene	500	ng/L
Fluoranthene	500	ng/L
Fluorene	500	ng/L
Indole	500	ng/L
Indeno(1,2,3-c,d)pyrene	500	ng/L
Naphthalene	500	ng/L
Perylene	500	ng/L
Phenanthrene	500	ng/L
Pyrene	500	ng/L
MECP Method 3400 - "THE DETERMINATION OF TRIFLURALIN IN WATER, EFFLUENT AND WASTEWATER BY HEXANE MICROEXTRACTION AND GAS CHROMATOGRAPHY - MASS SPECTROMETRY" (1L glass amber jar)		
PCB; total	20	ng/L
MECP Method 3302 – "THE DETERMINATION OF ARSENIC, SELENIUM AND ANTIMONY IN LIQUID INDUSTRIAL WASTE AND LANDFILL LEACHATES BY HYDRIDE-FLAMELESS ATOMIC ABSORPTION SPECTROMETRY (HYD-FAAS)" (500 ml PET) Influent and Effluent MDLs		
Arsenic	0.03 (0.001)	mg/L
Selenium	0.03 (0.001)	mg/L
MECP Method 3094 - "THE DETMRINATION OF METALS IN EFFLUENT, INDUSTRIAL WASTE AND LANDFILL LEACHATE BY INDUCTIVELY COUPLED PLASMA-ATOMIC EMISSION SPECTROSCOPY (ICP-AES)" Influent (500 ml PET)		
Aluminum	0.09	mg/L
Barium	0.02	mg/L
Beryllium	0.032	mg/L
Cadmium	0.026	mg/L
Calcium	0.026	mg/L
Chromium	0.018	mg/L
Cobalt	0.029	mg/L
Copper	0.023	mg/L
Iron	0.49	mg/L
Lead	0.08	mg/L
Magnesium	0.036	mg/L
Manganese	0.021	mg/L
Molybdenum	0.022	mg/L

Nickel	0.047	mg/L
Potassium	0.05	mg/L
Silver	0.072	mg/L
Sodium	0.2	mg/L
Strontium	0.024	mg/L
Titanium	0.016	mg/L
Vanadium	0.024	mg/L
Zinc	0.026	mg/L

MECP Method 3094 - "THE DETMRINATION OF METALS IN EFFLUENT, INDUSTRIAL WASTE AND LANDFILL LEACHATE BY INDUCTIVELY COUPLED PLASMA-ATOMIC EMISSION SPECTROSCOPY (ICP-AES) "Effluent (500 ml PET)

Aluminum	0.016	mg/L
Barium	0.001	mg/L
Beryllium	0.002	mg/L
Cadmium	0.002	mg/L
Calcium	0.007	mg/L
Chromium	0.003	mg/L
Cobalt	0.004	mg/L
Copper	0.002	mg/L
Iron	0.005	mg/L
Lead	0.02	mg/L
Magnesium	0.003	mg/L
Manganese	0.002	mg/L
Molybdenum	0.005	mg/L
Nickel	0.006	mg/L
Potassium	0.003	mg/L
Silver	0.004	mg/L
Sodium	0.01	mg/L
Strontium	0.001	mg/L
Titanium	0.001	mg/L
Vanadium	0.002	mg/L
Zinc	0.003	mg/L

Table S4. List of analytes, their methods, and method detection limits for Surface Waters

Parameter name	Practical Quantitation Limit	Units
MECP Method E3480 - "THE DETERMINATION OF POLYCLYCLIC AROMATIC HYDROCARBONS IN WATER BY GAS CHROMATOGRAPHY/MASS SPECTROMETRY" (1L glass amber jar)		
Phenanthrene	10	ng/L
Anthracene	10	ng/L
Fluoranthene	10	ng/L
Pyrene	20	ng/L
Benzo(a)anthracene	10	ng/L
Chrysene	10	ng/L
7,12-dimethylbenz(a)anthracene	10	ng/L
Benzo(b)fluoranthene	10	ng/L
Benzo(k)fluoranthene	10	ng/L
Benzo(e)pyrene	10	ng/L
Benzo(a)pyrene	10	ng/L
Perylene	1	ng/L
Indeno(1,2,3-c,d)pyrene	10	ng/L
Dibenzo(a,h)anthracene	20	ng/L
Benzo(g,h,i)perylene	20	ng/L
Naphthalene	20	ng/L
2-methylnaphthalene	10	ng/L
1-methylnaphthalene	10	ng/L
Acenaphthylene	10	ng/L
Acenaphthene	10	ng/L
Fluorene	10	ng/L
MECP Method E3459 - "THE DETERMINATION OF POLYCHLORINATED BIPHENYL (PCBS) CONGENERS IN AQUEOUS SAMPLES BY GC-HRMS" (1L glass amber jar)		
2-monochloroPCB(1)	TargetLynx software calculates the Limit of Detection (LOD), which is used as a guide in reporting method detection limits on a per sample basis, taking into consideration matrix effects, sample size, and surrogate recovery.	pg/L
4-monochloroPCB(3)		pg/L
2,2'-dichloroPCB(4)+2,6-dichloroPCB(10)		pg/L
2,3'-dichloroPCB(6)		pg/L
2,4'-dichloroPCB(8)		pg/L
4,4'-dichloroPCB(15)		pg/L
2,2',6-trichloroPCB(19)		pg/L
2,2',5-trichloroPCB(18)		pg/L
2,2',3-trichloroPCB(16)		pg/L
2,4',5-trichloroPCB(31)		pg/L
244'-triClPCB(28)+2'34-triClPCB(33)		pg/L
2,3,4'-trichloroPCB(22)		pg/L
3,4,4'-trichloroPCB(37)		pg/L
2,2',6,6'-tetrachloroPCB(54)		pg/L
2,2',5,5'-tetrachloroPCB(52)		pg/L
2,2',4,5'-tetrachloroPCB(49)		pg/L
2,2',3,5'-tetrachloroPCB(44)		pg/L
2,2',3,4-tetrachloroPCB(41)		pg/L
2,2',3,3'-tetrachloroPCB(40)		pg/L
2,4,4',5-tetrachloroPCB(74)		pg/L
2,3',4',5-tetrachloroPCB(70)		pg/L
2,3',4,4'-tetrachloroPCB(66)		pg/L
2,3,4,4'-tetrachloroPCB(60)		pg/L
3,4,4',5-tetrachloroPCB(81)		pg/L
3,3',4,4'-tetrachloroPCB(77)		pg/L
2,2',4,6,6'-pentachloroPCB(104)		pg/L
2,2',3,5',6-pentachloroPCB(95)		pg/L
PeClPCB(84)+PeCl(90)+PeCl(101)		pg/L
2,2',4,4',5-pentachloroPCB(99)		pg/L

2,3',4,4',6-pentachloroPCB(119)	pg/L
2,2',3',4,5-pentachloroPCB(97)	pg/L
2,2',3,4,5'-pentachloroPCB(87)	pg/L
2,2',3,4,4'-pentachloroPCB(85)	pg/L
2,3,3',4',6-pentachloroPCB(110)	pg/L
2',3,4,4',5-pentachloroPCB(123)	pg/L
2,3',4,4',5-pentachloroPCB(118)	pg/L
2,3,4,4',5-pentachloroPCB(114)	pg/L
2,3,3',4,4'-pentachloroPCB(105)	pg/L
3,3',4,4',5-pentachloroPCB(126)	pg/L
2,2',4,4',6,6'-hexachloroPCB(155)	pg/L
2,2',3,5,5',6-hexachloroPCB(151)	pg/L
2,2',3,3',5,6'-hexachloroPCB(135)	pg/L
2,2',3,4',5',6-hexachloroPCB(149)	pg/L
22'44'55'(153)+23'44'5'6-HxClPCB(168)	pg/L
2,2',3,4,5,5'-hexachloroPCB(141)	pg/L
2,2',3,4,4',5-hexachloroPCB(137)	pg/L
2,2',3,4,4',5'-hexachloroPCB(138)	pg/L
22'33'45(129)+233'44'6-HxClPCB(158)	pg/L
2,2',3,3',4,4'-hexachloroPCB(128)	pg/L
2,3',4,4',5,5'-hexachloroPCB(167)	pg/L
2,3,3',4,4',5-hexachloroPCB(156)	pg/L
2,3,3',4,4',5'-hexachloroPCB(157)	pg/L
3,3',4,4',5,5'-hexachloroPCB(169)	pg/L
2,2',3,4',5,6,6'-heptachloroPCB(188)	pg/L
2,2',3,3',5,5',6-heptachloroPCB(178)	pg/L
2,2',3,4',5,5',6-heptachloroPCB(187)	pg/L
2,2',3,4,4',5',6-heptachloroPCB(183)	pg/L
2,2',3,3',4,5,6'-heptachloroPCB(174)	pg/L
2,2',3,3',4',5,6-heptachloroPCB(177)	pg/L
2,2',3,3',4,4',6-heptachloroPCB(171)	pg/L
22'344'55'(180)+233'4'55'6-HpClPCB(193)	pg/L
2,3,3',4,4',5',6-heptachloroPCB(191)	pg/L
2,2',3,3',4,4',5-heptachloroPCB(170)	pg/L
2,3,3',4,4',5,5'-heptachloroPCB(189)	pg/L
2,2',3,3',5,5',6,6'-octachloroPCB(202)	pg/L
2,2',3,3',4,5',6,6'-octachloroPCB(201)	pg/L
2,2',3,3',4,5,6,6'-octachloroPCB(200)	pg/L
2,2',3,3',4,5,5',6'-octachloroPCB(199)	pg/L
2,2',3,4,4',5,5',6-octachloroPCB(203)	pg/L
2,2',3,3',4,4',5,5'-octachloroPCB(194)	pg/L
2,3,3',4,4',5,5',6-octachloroPCB(205)	pg/L
22'33'455'66'-nonachloroPCB(208)	pg/L
22'33'44'566'-nonachloroPCB(207)	pg/L
22'33'44'55'6-nonachloroPCB(206)	pg/L
DecachloroPCB(209)	pg/L
Monochlorobiphenyls;total	pg/L
Dichlorobiphenyls;total	pg/L
Trichlorobiphenyls;total	pg/L
Tetrachlorobiphenyls;total	pg/L
Pentachlorobiphenyls,total	pg/L
Hexachlorobiphenyls;total	pg/L
Heptachlorobiphenyls;total	pg/L
Octachlorobiphenyls;total	pg/L
Nonachlorobiphenyls;total	pg/L
PCB congeners;total	pg/L

MECP Method 3089 – "THE DETERMINATION OF ARSENIC, SELENIUM AND ANTIMONY IN LIQUID INDUSTRIAL WASTE AND LANDFILL LEACHATES BY HYDRIDE-FLAMELESS ATOMIC ABSORPTION SPECTROMETRY (HYD-FAAS)" (500 ml PET)

Arsenic	0.001	mg/L
Selenium	0.001	mg/L

MECP Method 3474 - "THE DETERMINATION OF TRACE METALS IN SURFACE AND GROUND WATERS BY DYNAMIC CELL (DRC) INDUCTIVELY COUPLED PLASMA-MASS SPECTROMETRY (ICP-MS)" (500 ml PET)

Aluminum	0.016	mg/L
Barium	0.001	mg/L
Beryllium	0.002	mg/L
Cadmium	0.002	mg/L
Calcium	0.007	mg/L
Chromium	0.003	mg/L
Cobalt	0.004	mg/L
Copper	0.002	mg/L
Iron	0.005	mg/L
Lead	0.02	mg/L
Magnesium	0.003	mg/L
Manganese	0.002	mg/L
Molybdenum	0.005	mg/L
Nickel	0.006	mg/L
Potassium	0.003	mg/L
Silver	0.004	mg/L
Sodium	0.01	mg/L
Strontium	0.001	mg/L
Titanium	0.001	mg/L
Vanadium	0.002	mg/L
Zinc	0.003	mg/L

Table S5: Viability and viable cell count (mean ± standard deviation) of the hemocytes of the spike mussel (*Eurynia dilatata*) used for *in vitro* toxicity testing, as assessed by hemocytometry.

Site	Sample collection	Number of replicates	Viability (%)		Viable Cell Count (cells/mL)	
			T=0	T= 4h	T=0	T=4 h
WWTP A&B	November 2014	4	97 ± 1.0	76 ± 8.9	$1.5 \cdot 10^6 \pm 4.72 \cdot 10^5$	$2.0 \cdot 10^5 \pm 1.59 \cdot 10^5$
WWTP C	June 2015	8	98 ± 1.3	77 ± 11.8	$7.1 \cdot 10^5 \pm 3.61 \cdot 10^5$	$2.3 \cdot 10^5 \pm 1.18 \cdot 10^5$
WWTP D	June 2015	4	94 ± 2.3	70 ± 28.1	$8.7 \cdot 10^5 \pm 4.93 \cdot 10^5$	$2.1 \cdot 10^5 \pm 1.54 \cdot 10^5$
WWTP C	October 2015	8	96 ± 2.3	75 ± 10.2	$8.3 \cdot 10^5 \pm 3.46 \cdot 10^5$	$2.8 \cdot 10^5 \pm 1.82 \cdot 10^5$
WWTP D	October 2015	8	94 ± 2.2	76 ± 8.1	$8.1 \cdot 10^5 \pm 2.19 \cdot 10^5$	$1.5 \cdot 10^5 \pm 5.70 \cdot 10^4$

Table S6: Concentration of compounds and nutrients detected in the Hamilton Harbour Area of Concern including Wastewater Treatment Plant (WWTP) A and B, and surrounding surface waters.

	Site/WWTP	WWTP A		WWTP B		West End Index Station		Hamilton Harbour Index Station		Windermere Arm Index Station	
	Sample type	Effluent		Effluent		Surface		Surface		Surface	
	Date	Nov-14		Nov-14		Jul-14		Jul-14		Jul-14	
PPCPs (ng/L)	Bezafibrate	107.3		34.3		2.0	<	2.0	<	2.0	<
	Bisphenol A	79.7		475.3		20.0	<	31.0		20.0	<
	Caffeine	360.0		187.0		10.0	<	10.0	<	10.0	<
	Carbamazepine	306.3		277.3		43.0		42.0		49.0	
	Ciprofloxacin	66.3		82.7		5.0	<	5.0	<	5.0	<
	DEET	18.0		31.0		30.0		120.0		150.0	
	Diclofenac	830.0		517.0		5.0	<	5.0	<	5.0	<
	Erythromycin	8.4		25.1		1.3		1.0	<	1.3	
	Gemfibrozil	4.0	<	5.3		2.0	<	2.0	<	2.0	<
	Glyburide	3.3		10.7		1.0	<	1.0	<	1.0	<
	Ibuprofen	40.0	<	40.0	<	20.0	<	20.0	<	20.0	<
	Indomethacin	18.0		15.0		2.0	<	2.0	<	2.0	<
	Ketoprofen	22.7		45.3		2.2		3.2		2.2	
	Lidocaine	183.0		138.3		13.0		11.0		17.0	
	Naproxen	20.0	<	20.0	<	10.0	<	10.0	<	10.0	<
	Sulfamethazine	4.0	<	352.7		2.1		2.1		2.1	
	Sulfamethoxazole	890.0		457.3		81.0		90.0		98.0	
	Triclocarban	6.0		4.7		0.8		2.3		1.1	
	Triclosan	40.0	<	40.0	<	20.0	<	20.0	<	20.0	<
	Trimethoprim	239.3		143.0		13.0		18.0		14.0	
PCBs(pg/L)	PCB77	0.55	<	1.14	<	0.78	<	1.90	<	1.4	<
	PCB81	0.45	<	0.26	<	0.31	<	1.30		0.2	<
	PCB105	3.70	<	17.33	#	5.20	<	9.20	<	17	<
	PCB114	0.29	<	1.13	#	0.26	<	0.60	<	0.95	<
	PCB118	12.33	<	52.00	#	18.00	<	27.00	<	60	<
	PCB123	0.93	<	2.33	<	1.90	<	2.40	<	5.4	<
	PCB126	0.27	<	0.21	<	0.14	<	0.46		0.28	
	PCB156	0.97	<	4.90	#	3.10		5.00		12.0	
	PCB157	0.26	<	1.01	#	0.59	<	0.87	<	2.0	
	PCB167	0.35	<	1.83	#	1.40	<	2.30		4.6	
	PCB189	0.11	<	0.28	#	0.63	<	0.84		2.2	
Metals (mg/L)	Nickel	0.006	<	0.006	<	1.7	*	1.7	*	1.8	*
NPs (ng/L)	4-Nonylphenol Monoethoxylate	20	<	20	<	20	<	20	<	20	<
	4-Nonylphenol Diethoxylate	5	<	5	<	5	<	5	<	5	<
	4-Nonylphenol	80.3		46.7		28.0		24.0		20.0	*
Dioxins/Furans (pg/L)	Octachlorofuran	0.357	<	0.423	<	0.24	<	0.4	<	0.26	<
	Octachlorodioxin	0.76	<	1.7	<	4.5		3.5		4.1	
	1234678-heptachlorodioxin	0.243	<	0.420	<	0.71	<	0.74	<	0.68	<
Nutrients (mg/L)	Ammonia (NH_3)+ ammonium (NH_4+)	0.055		0.127		0.083		0.089		0.140	
	Nitrite (NO_2-)	11.653		0.181		0.106		0.111		0.124	
	Nitrate (NO_3-)+ nitrite (NO_2-)	5.981		15.967		2.350		2.560		2.710	
	Phosphate (PO_4)	13.359		0.212		0.003	<	0.002	<	0.004	<
pH	pH	7.7		7.6		7.7		7.8		7.8	
Conductivity (µS/cm)	Conductivity	1123		1780		760		774		786	
Alkalinity (mg/L $CaCO_3$)	Alkalinity	82		105		104		102		102	
TSD (mg/L)	Suspended solids	0.8		4.433		5.4		4.9		5.7	
BOD	Biochemical oxygen demand	1.15		4.67		2.33		8.03		10.27	

Annotations: < indicates the method detection limit (MDL) or values measured below the MDL; # indicates values where one of the two replicates was below the MDL; * indicates samples that were improperly preserved.

Table S7: Concentration of compounds and nutrients detected in the Toronto and Region Area of Concern, including Wastewater Treatment Plant (WWTP) C and D and surrounding surface waters.

	Site/WWTP	WWTP C				Diffuser	River Plume	Humber Bay Index Station	WWTP D				Toronto Harbour Index Station	
	**Sample type**	**Influent**	**Effluent**	**Influent**	**Effluent**	**Surface**	**Surface**	**Surface**	**Influent**	**Effluent**	**Influent**	**Effluent**	**Surface**	**Surface**
	**Date**	**Jun-15**	**Jun-15**	**Oct-15**	**Oct-15**	**Jun-15**	**Jun-15**	**Jun-15**	**Jun-15**	**Jun-15**	**Oct-15**	**Oct-15**	**Jun-15**	**Jul-15**
**PPCPs (ng/L)**	**Bezafibrate**	80.4	37.7	101.6	46.3	2 <	2 <	2 <	74.8	23.8	132.8	46.7	2 <	2 <
	**Bisphenol A**	3296	163.3	872	-	20 <	20 <	20 <	259.6	75.4	186.4	370.5	30	20 <
	**Caffeine**	22800	269.3	5040	400	150.0	44	33	456.0	166.3	2556	63.5	79	120
	**Carbamazepine**	940	370.0	255.2	501.2	22.0	3.8	3.5	84.0	205.2	210.8	359.7	4.2	5.4
	**Ciprofloxacin**	393.2	91.6	255.6	172.7	5 <	5 <	5 <	112.4	29.1	270.8	117.4	5 <	5 <
	**DEET**	2160	18.5	616	6.5	99	12	11	1200	91.8	724	19.9	17.0	31.0
	**Diclofenac**	944.0	709.3	688.0	547.5	40.0	5 <	5 <	424.0	273.3	520.0	506.7	5 <	5 <
	**Erythromycin**	14.3	100.9	14.7	33.5	1.2	1 <	1 <	8.0 <	8.1	12.8	10.4	1 <	1 <
	**Gemfibrozil**	74.4	4.0 <	47.6	12.6	2 <	2 <	2 <	37.4	4.0 <	39.3	6.8	2 <	2 <
	**Glyburide**	34.6	67.1	8.0 <	9.6	1 <	1 <	1 <	8.0 <	2.1	8.0 <	3.3	1 <	1 <
	**Ibuprofen**	4040	40 <	3996	40 <	20 <	20 <	20 <	612	40 <	1400	40 <	20 <	20 <
	**Indomethacin**	23.8	13.9	22.3	25.7	2 <	2 <	2 <	16.0 <	5.7	18.3	19.3	2 <	2 <
	**Ketoprofen**	119.2	28.5	101.6	55.4	2 <	2 <	2 <	86.4	20.0	109.2	36.1	2 <	9.5
	**Lidocaine**	314.8	419.7	162.4	384.7	19.0	2.0	2.3	145.2	211.5	147.6	271.2	2.2	2.5
	**Naproxen**	5760.0	76.0	5480.0	173.3	10.0 <	10.0 <	10 <	238.8	51.8	115.6	53.4	10 <	10 <
	**Sulfamethazine**	23.2	4.0 <	38.8	4.8	2.0 <	2.0 <	2 <	16.0 <	4.0 <	16.0 <	4.0 <	2 <	2 <
	**Sulfamethoxazole**	16.0 <	238.3	460.0	214.7	20.0	5.9	5.5	16.0 <	231.7	275.6	463.7	6.0	7.5
	**Triclocarban**	188.4	8.5	191.2	48.5	1.4	0.5 <	0.5 <	60.4	13.6	132.0	26.3	0.5 <	0.5 <
	**Triclosan**	1656.0	40.0 <	860.0	69.6	20.0 <	20.0 <	20 <	424.0	40.0 <	160.0 <	103.0	20.0 <	20.0 <
	**Trimethoprim**	133.6	132.7	90.4	147.3	2.1	2.0 <	2 <	50.0	109.4	108.4	176.3	2.0 <	2.0 <
**PCBs (pg/L)**	**PCB77**	52.0	1.70 <	19	1.9 <	0.72 <	0.43 <	2.2 <	8.6	0.87 <	8.5	1.20 <	0.87 <	0.54 <
	**PCB81**	7.2	0.19 <	0.75	0.18 <	0.14 <	0.18 <	1.5	0.51 <	0.13 <	0.34 <	0.15 <	0.25 <	0.19 <
	**PCB105**	240.0	40.0	680	38 <	7.4 <	3.1 <	6.6 <	190.0	11.00 <	110.0	15.00 <	5.30 <	3.95 <
	**PCB114**	14.0	2.3	33	2 <	0.44 <	0.25 <	0.5 <	9.8	0.79 <	6.3	0.84 <	0.29 <	0.34 <
	**PCB118**	620.0	120.0	1600	110 <	18.0 <	8.1 <	26.0 <	500.0	32.0 <	290.0	47.0 <	16.0 <	12.0 <
	**PCB123**	16.0	3.0 <	44	4 <	1.6 <	0.92 <	3.10 <	8.7	1.7 <	9.8 <	2.2 <	1.8 <	1.5 <
	**PCB126**	1.7	0.19	1.2	0.17	0.12 <	0.12 <	0.82	0.59	0.12 <	0.59	0.12 <	0.27	0.2 <
	**PCB156**	48.0	6.40	230	8.2	3.0 <	1.1 <	1.90 <	52.0	2.6 <	29.0	4.2 <	1. <	0.97 <
	**PCB157**	11.0	1.40 <	42	1.9	0.81 <	0.43 <	0.35 <	10.0	0.68 <	5.3	0.9 <	0.43 <	0.29 <
	**PCB167**	17.0	2.40	67	3.1 <	1.4 <	0.7 <	0.85 <	17.0	1.1 <	8.4	1.5 <	0.75 <	0.57 <
	**PCB189**	2.9	0.26 <	10	0.4	0.41 <	0.19 <	0.19 <	2.2	0.18 <	1.4	0.17 <	0.13 <	0.15 <
**Metals**	**Nickel (mg/L)**	0.02 <	0.006 <	0.02 <	0.006 <	0.04 <	0.04 <	0.04 <	0.02 <	0.006 <	0.02 <	0.006 <	0.04 <	1.80
**NPs (ng/L)**	**4-Nonylphenol Monoethoxylate**	1600	20.0 <	7200	25.7 #	20 <	20 <	20 <	4400	20 <	5600	163.3	20 <	20 <
	**4-Nonylphenol Diethoxylate**	90	5.0 <	610	19.3	5 <	5 <	5 <	540	5 <	530	89.3	5 <	5 <
	**4-Nonylphenol**	4100	85.3	2300	47.7	22*	20*	20*	5100	94	4000	270.0	20*	28*
**Dioxins and Furans (pg/L)**	**Octachlorofuran**	2.2	0.19 <	1.6	0.25 <	0.61 <	0.26 <	0.63	2.5	0.17 <	1.6	0.17 <	0.32 <	0.21 <
	**Octachlorodioxin**	42	1.8 <	30	1.9	7.4	2.1 <	2.2	28	2.3	200	1.2 <	1.8	1.075 <
	**1234678-heptachlorodioxin**	5.6	0.28 <	3.5	0.39 <	1.2 <	0.57 <	0.53 <	3.9	0.38 <	15	0.29 <	0.42 <	0.31
**Nutrients (mg/L)**	**Ammonia (NH_3)+ ammonium (NH_4+)**	13.4	0.101	27.1	0.312	0.093	0.058	0.044	18.0	0.465	27.6	6.707	0.058	0.041
	**Nitrite (NO_2-)**	0.024	0.437	0.040	1.577	0.03	0.005	0.004 <	0.146	0.120	0.026	0.790	0.007	0.014
	**Nitrate (NO_3-)+ nitrite (NO_2-)**	0.02 <	7.720	0.02 <	16.63	1.31	0.319	0.291	0.061	14.667	0.02 <	9.277	0.316	0.275
	**Phosphate (PO_4)**	0.730	0.121	2.150	0.668	0.033	0.008	0.004 <	1.090	0.517	2.480	0.401	0.005	0.003 <
**pH**	**pH**	7.2	7.9	7.3	7.5	8.0	8.2	8.2	7.6	7.9	7.6	7.8	8.1	8.3
**Conductivity (µS/cm)**	**Conductivity**	1370	1240	1230	1047	497	332	319	1250	1170	1020	915	348	365
**Alkalinity (mg/L $CaCO_3$)**	**Alkalinity**	241	155	253	101	107	90	88	246	125	230	122	90	94
**TSD (mg/L)**	**Suspended solids**	171	5.67	84.1	8.47	21.9	4.6	1.3 <	317	11.1	162	4.13	1.3 <	1.3 <
**BOD**	**Biochemical oxygen demand**	2.7	3.1	1.2	1.6	1.4	1.1	1.6	6.27	295	265	346	205	3.4

Annotations: < indicates the method detection limit (MDL) or values measured below the MDL; # indicates values where one of the two replicates was below the MDL; * indicates samples that were improperly

Method validation – freshwater mussels – ethanol and hydrogen peroxide

Methods

Freshwater mussels (*Eurynia dilatata*) and hemocytes were treated as described in the main article. For method validation, cells were exposed to 25% PBS (control), 0.1% ethanol (solvent control), 0.125 mM hydrogen peroxide (positive control 1) or 0.25 mM hydrogen peroxide (positive control 2) (n=4) in 96-well microplates for 4 h at 20°C in the dark, under gentle agitation (150 rpm). We combined 150 µL of each control solution to 150 µL of hemolymph.

At the end of the exposure, the viability and cell density of hemolymph from each treatment was assessed by flow cytometry. Furthermore, the controls were evaluated for the comet assay. Cells were handled as described in the main article and for each control, pictures of 50 cells were processed using a comet application in Northern Eclipse. The percentage of DNA in the comet tail (% DNA in tail) was selected as the most reliable and meaningful measurement of DNA damage.

Results

The DNA damage in hemocytes exposed to 0.125 mM and 0.25 mM H_2O_2 increased by up to a factor of two after 4 hours, in comparison with the 25% PBS control ($p \leq 0.02$, Figure S2B). In contrast, exposure to 0.1% EtOH did not significantly alter DNA damage. As the final concentration of ethanol in the extracts of all sample sites and dilutions was 0.1% EtOH, further experiments were compared to the 0.1 % EtOH (solvent) and 0.25 mM H_2O_2 (positive) controls.

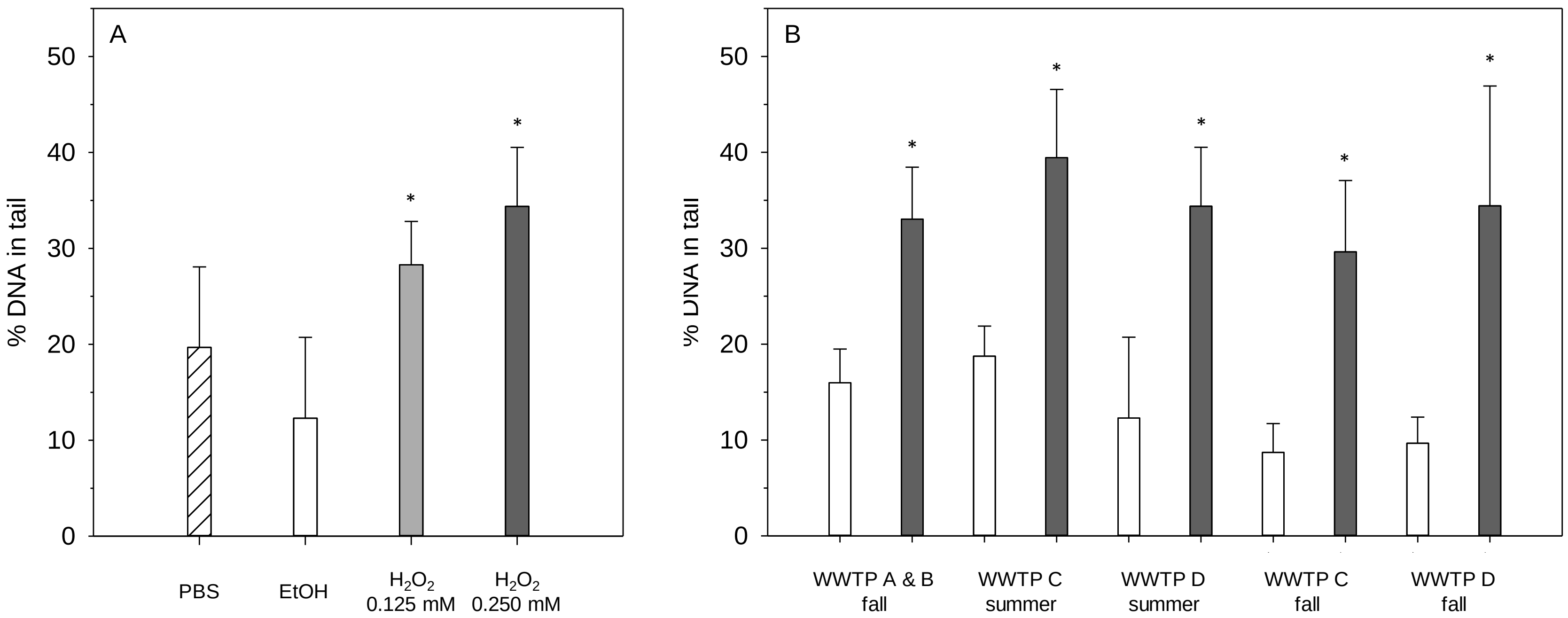

Figure S1: A) DNA damage (mean ± SD, n=4; expressed as % tail DNA) in control (PBS), solvent control (0.1% EtOH), and hydrogen peroxide (H_2O_2) positive controls in the Spike mussel (*Eurynia dilatata*) after a 4-h exposure. Asterisk (*) indicate significant difference from the ethanol controls ($p < 0.05$) B) DNA damage of the hemocytes of the freshwater mussel (*Eurynia dilatata*) after a 4-h exposure to the solvent control [ETOH: 0.1% (white)] or the positive control [H_2O_2: 0.25 mM hydrogen peroxide (grey)]. Asterisk (*) indicate a significant difference from their respective ethanol control ($p < 0.01$; α= 0.05/5 adjusted for multiple comparisons).

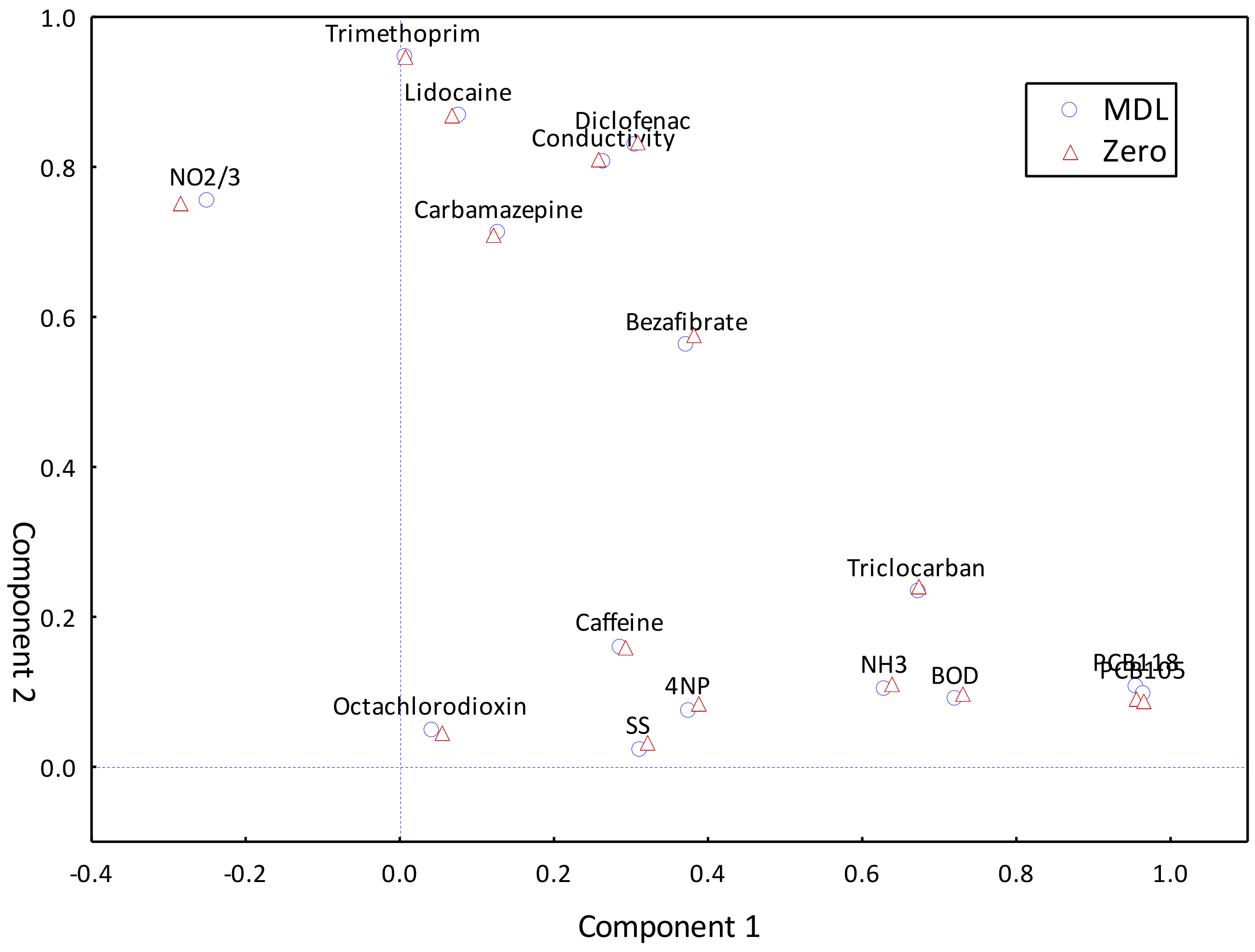

Figure S2. Principal component scores of measured contaminants in water or effluent samples, using either zero or the Method Detection Limit (MDL) as replacement values. The percent difference in scores from the Principal Component Analyses differed only by an average of 0.94% when using MDLs (O) or zero (Δ) as replacement values. Hence, the method used to replace observations below MDLs was inconsequential to any interpretation of the PCA.

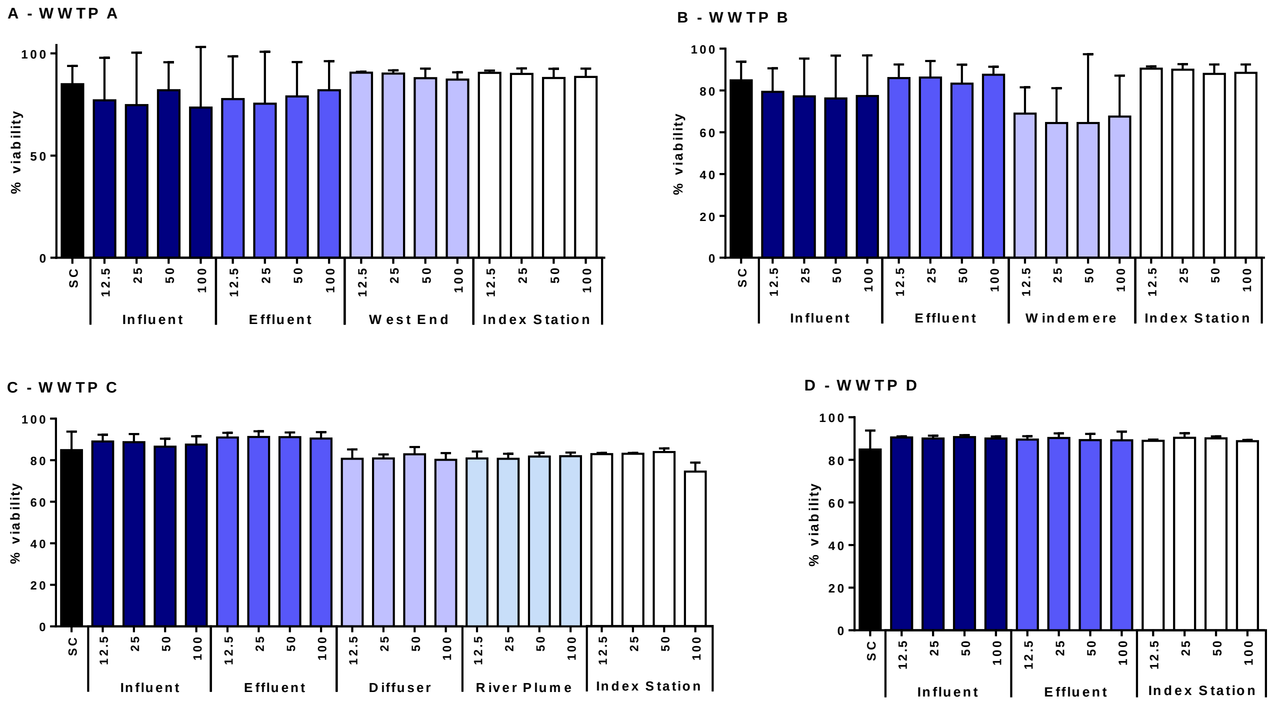

Figure S3: Rainbow trout leucocyte viability, expressed as % of viable leucocytes in the cell population (mean ± standard deviation, *n=3*), after a 24-h exposure to increasing concentration of organic fraction of influent, effluent and surface water samples (expressed as % of viable leucocytes in the cell population) from sites in the Hamilton Harbour AOC (A, B) and the Toronto and Region AOC (C, D). There were no significant differences in leucocyte viability between control (RPMI, data not shown) and 0.1% EtOH treatments.
